# Development of Novel Quinoline-Based Sulfonamides as Selective Cancer-Associated Carbonic Anhydrase Isoform IX Inhibitors

**DOI:** 10.3390/ijms222011119

**Published:** 2021-10-15

**Authors:** Moataz Shaldam, Alessio Nocentini, Zainab M. Elsayed, Tamer M. Ibrahim, Rofaida Salem, Ramadan A. El-Domany, Clemente Capasso, Claudiu T. Supuran, Wagdy M. Eldehna

**Affiliations:** 1 Department of Pharmaceutical Chemistry, Faculty of Pharmacy, Kafrelsheikh University, Kafrelsheikh 33516, Egypt; dr_moutaz_986@pharm.kfs.edu.eg (M.S.); Tamer_Mohamad@pharm.kfs.edu.eg (T.M.I.); rofida_salem@pharm.kfs.edu.eg (R.S.); 2 Department of NEUROFARBA, Section of Pharmaceutical and Nutraceutical Sciences, University of Florence, Polo Scientifico, Via U. Schiff 6, Sesto Fiorentino, 50019 Firenze, Italy; alessio.nocentini@unifi.it; 3 Scientific Research and Innovation Support Unit, Faculty of Pharmacy, Kafrelsheikh University, Kafrelsheikh 33516, Egypt; zienabmohammed9@gmail.com; 4 Department of Microbiology and Immunology, Faculty of Pharmacy, Kafrelsheikh University, Kafrelsheikh 33516, Egypt; Ramadan_Eldomany@pharm.kfs.edu.eg; 5 Institute of Biosciences and Bioresources, Italian National Research Council (CNR)CNR, Via Pietro Castellino 111, 80131 Napoli, Italy; clemente.capasso@ibbr.cnr.it

**Keywords:** sulfonamides, 4-anilinoquinoline, carbonic anhydrase IX inhibitors, anticancer agents, hypoxic tumors

## Abstract

A new series of quinoline-based benzenesulfonamides (**QBS**) were developed as potential carbonic anhydrase inhibitors (CAIs). The target **QBS** CAIs is based on the 4-anilinoquinoline scaffold where the primary sulphonamide functionality was grafted at C4 of the anilino moiety as a zinc anchoring group (**QBS** **13a**–**c**); thereafter, the sulphonamide group was switched to *ortho*- and *meta*-positions to afford regioisomers **9a**–**d** and **11a**–**g**. Moreover, a linker elongation approach was adopted where the amino linker was replaced by a hydrazide one to afford **QBS** **16**. All the described **QBS** have been synthesized and investigated for their CA inhibitory action against *h*CA I, II, IX and XII. In general, *para*-sulphonamide derivatives **13a**–**c** displayed the best inhibitory activity against both cancer-related isoforms *h*CA IX (*K*_I_s = 25.8, 5.5 and 18.6 nM, respectively) and *h*CA XII (*K*_I_s = 9.8, 13.2 and 8.7 nM, respectively), beside the excellent *h*CA IX inhibitory activity exerted by *meta*-sulphonamide derivative **11c** (*K*_I_ = 8.4 nM). The most promising **QBS** were further evaluated for their anticancer and pro-apoptotic activities on two cancer cell lines (MDA-MB-231 and MCF-7). In addition, molecular docking simulation studies were applied to justify the acquired CA inhibitory action of the target QBS.

## 1. Introduction

Carbonic anhydrases (CA) are metalloenzymes that catalyze the reversable interconversion between carbon dioxide and bicarbonate ion reaction [1]. Human carbon anhydrases (*h*CAs) belong to the α-CA family, one of the eight discovered families of carbon anhydrases [2]. Only twelve *h*CAs (*h*CAs I–VII, *h*CA IX and *h*CAs XII–XIV) out of the sixteen isozymes discovered exhibit catalytic action [3]. CAs are involved in both biological and pathological processes such as homeostasis of pH, respiration, bone resorption, epilepsy, tumorigenicity and obesity [4,5]. The role of CAs in various diseases has been confirmed and several *h*CAs isoforms are, therefore, valuable targets for designing inhibitors with clinical applications, such as anti-glaucoma, antiepileptic, anti-obesity, anticancer, etc. [6,7,8,9,10,11]. 

Tumor progression induces a hypoxic environment that triggers extracellular acidosis as a result of anaerobic glycolysis in the tumor cell and this drop in pH further stimulates the tumor growth [12,13]. *h*CA IX and *h*CA XII isoforms are generally called “cancer-associated” CA isoforms. The *h*CA IX isoform is upregulated in nearly all hypoxic tumors so that it can maintain the intracellular pH and promote the acidic extracellular environment required for promoting tumor growth and metastasis [13,14]. In addition, *h*CA IX is involved in cell proliferation as well as cell to cell communication. Overexpression of *h*CA IX isoform is strongly correlated with poor prognosis in many cancers [15]. Moreover, *h*CA XII is also accompanied with many tumor types, but it is less associated to a hypoxic tumor when compared with *h*CA IX [16].

**Figure 1:**
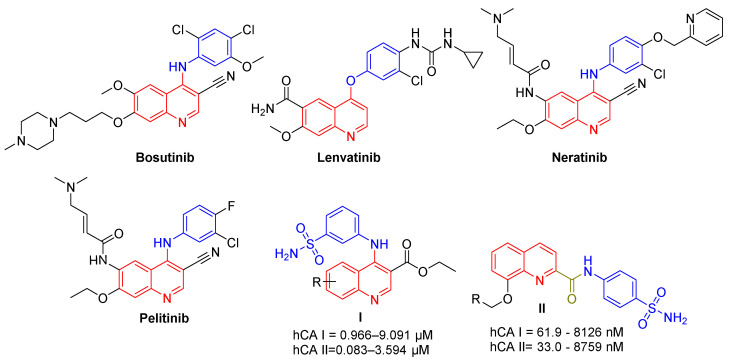
Chemical structure of clinically approved quinoline-based drugs; Bosutinib, Lenvatinib and Neratinib, and the investigational drug Pelitinib, as well as CAIs quinolines **I** and **II**.

Quinoline-based small molecules have been reported to exhibit diverse biological activities including anticancer activity [17]. Bosutinib and Lenvatinib (Figure 1) are quinoline-based kinase inhibitors approved for chronic myelogenous leukemia and thyroid cancer, respectively [18]. Moreover, Bosutinib has been investigated in clinical trials for the treatment of breast cancer [19,20,21]. In addition, Neratinib (Figure 1), a tyrosine kinase inhibitor, is FDA approved for metastatic HER2-positive breast cancer [22], whereas, Pelitinib (Figure 1) is a second generation irreversible epidermal growth factor receptor tyrosine kinase (EGFR TK) inhibitor that is currently examined in phase II clinical trials for the treatment of non-small cell lung cancer and colorectal cancer [23]. 

To the best of our knowledge, few studies have reported on the development of quinoline-based sulfonamides as carbonic anhydrase inhibitors. In 2019, a novel series of 3-(quinolin-4-ylamino)benzenesulfonamides was reported as *h*CA I and II inhibitors. These derivatives, with general structure **I** (Figure 1), displayed weak inhibitory activity against the *h*CA I isoform (*K*_I_ range: 0.96 μM–9.09 μM) and moderate activity toward the *h*CA II isoform (*K*_I_ range: 83.3 nM–3.59 μM) [24]. In the same year, another series of quinoline-2-carboxamides (general structure **II**, Figure 1) was reported as a novel *h*CA inhibitor [25]. Quinolines **II** exerted moderate inhibitory activity against *h*CA II and IV isoforms, whereas they did not display any significant activity against the cancer-associated *h*CA IX isoform.

**Figure 2:**
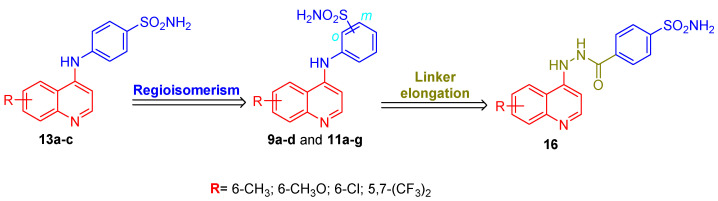
Design of target QBS (**9a**–**d**, **11a**–**g**, **13a**–**c** and **16**).

Resuming the efforts to create effective *h*CA IX and *h*CA XII inhibitors, here we describe the design and synthesis of novel 4-aminoquinoline-based sulfonamides (Figure 2). The design of the herein reported target QBS CAIs is based on the incorporation of 6-substituted quinoline as a lipophilic tail. The fused lipophilic quinoline tails are anticipated to achieve significant hydrophobic interactions within the roomier *h*CA IX and XII binding sites. The substitutions on a quinoline ring including -CH_3_, -OCH_3_, -Cl and di-CF_3_ span different electronic properties. Furthermore, different positional isomers “*ortho*, *meta* and *para*” of aminobenzenesulfonamide moieties were incorporated to provide the target series **QBS 9a**–**d**, **11a**–**g** and **13a**–**c**, Figure 2. In addition, a linker elongation approach was adopted where the amino linker was replaced by a hydrazide one to afford **QBS 16**.

All the herein designed quinolines were synthesized, characterized and explored for their CA inhibitory action against *h*CA I, II, IX and XII. Then, the anti-proliferation and the apoptosis induction effects of the most efficient *h*CA IX inhibitors were in vitro investigated. Molecular docking simulation studies were applied to justify the CA inhibitory action of the target quinolines.

## 2. Results and Discussion

### 2.1. Chemistry

**Scheme 1:**
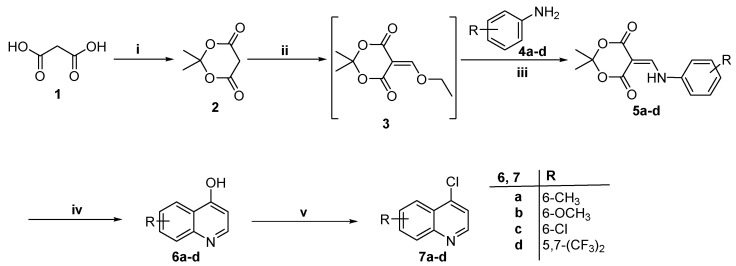
Reagent and conditions: (**i**) Acetic anhydride, conc. H_2_SO_4_, acetone RT; (**ii**) Triethyl orthoforamte, reflux 3 h; (**iii**) DMF, reflux 2 h; (**iv**) Diphenyl ether, Microwave at 250 °C, 10–15 min; (**V**) POCl_3_, 90 °C, 4 h.

**Scheme 2:**
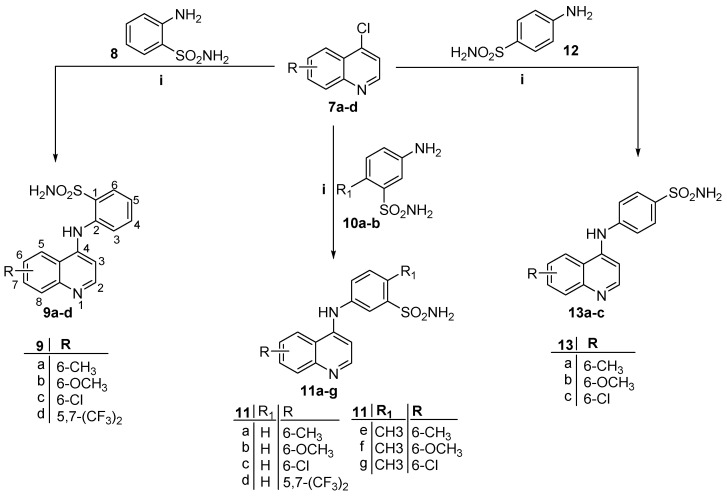
Reagent and conditions: (**i**) Isopropanol, catalytic HCl, reflux 2 h.

**Scheme 3:**
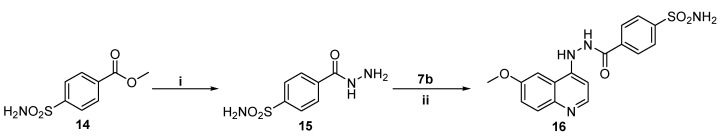
Reagent and conditions: (**i**) Isopropyl alcohol, NH_2_NH_2_.H_2_O, reflux 3 h; (**ii**) Isopropanol, catalytic HCl, reflux 2 h.

The preparation of **QBS** (**9a**–**d**, **11a**–**g**, **13a**–**c** and **16**) is illustrated in Scheme 1, Scheme 2 and Scheme 3. 

Meldrum’s acid **2** was prepared from malonic acid and acetone according to the reported procedures [26]. Then, heating **2** with triethyl orthoformate under gentile reflux followed by the addition of substituted anilines **4a**–**d**, afforded 5-(phenylaminomethylene) meldrum’s acids **5a**–**d**. The cyclization of **5a**–**d** was achieved via microwave irradiation for 10–15 min at 250 °C to produce 6-substiuted 4-hydroxyquinolines **6a**–**d** in 66–70% yield [27]. Heating Meldrum’s acid at temperatures greater than 200 °C leads to a pericyclic reaction that produces the highly reactive ketene, which was subjected to nucleophilic addition by the phenyl ring [28,29]. Thereafter, 6-substiuted 4-hydroxyquinolines **6a**–**d** were converted to the corresponding 4-chloroquinoline derivatives **7a**–**d** in 70–75% yield by heating with an excess of phosphorus oxychloride for 4 h. (Scheme 1). 

In Scheme 2, the 4-chloroquinoline derivatives **7a**–**d** were reacted with different aminobenzenesulfonamide derivatives (**8**, **10a**–**b** and **12**) in refluxing isopropanol in the presence of a catalytic amount of HCl to furnish the corresponding target **QBS** derivatives (**9a**–**d**, **11a**–**g** and **13a**–**c**) with a 65–78% yield. 

The methyl 4-sulfamoylbenzoate **14** was subjected to hydrazinolysis to afford hydrazide **15**, through refluxing with hydrazine hydrate in isopropyl alcohol. Thereafter, 6-methoxy-4-chloroquinoline **7b** was reacted with 4-(hydrazinecarbonyl)benzenesulfonamide **15** in refluxing isopropanol to furnish the corresponding target **QBS 16** with a 69% yield (Scheme 3). 

Postulated structures for the newly synthesized **QBS** compounds were in full agreement with their spectral analyses data (Supporting Information).

### 2.2. Biological Evaluation

#### 2.2.1. Carbonic Anhydrases Inhibition

The potential CA inhibition activity of the newly synthesized **QBS** (**9a**–**d**, **11a**–**g**, **13a**–**c** and **16**) were assessed applying the stopped flow carbon dioxide hydrase assay [30] for both the ubiquitous CA isoforms *h*CA I and II, and the cancer-related isoforms *h*CA IX and XII. The tested CA isoforms were suppressed to varying degrees by the herein reported **QBS**, and the inhibition data are displayed in Table 1. 

The cytosolic *h*CA I isoform was inhibited by all the quinoline-based sulfonamides reported in this study with inhibition constants (*K*_I_s) ranging from low nanomolar to low micromolar concentrations, between 55.4 nM and 1.56 μM, apart from 4-methyl-3-sulfonamide bearing counterparts **11e**–**g**, which exerted lower inhibitory actions (*K*_I_ = 3.498, 2.256 and 4.521 μM, respectively). In particular, all the *para*-sulphonamide analogous **13a**–**c** and **16** were found to be the most effective *h*CA I inhibitors with two-digit nanomolar inhibition constants (*K*_I_s = 78.4, 92.1, 55.4 and 81.4 nM, respectively). Moreover, *meta*-sulphonamide analogous **11a**–**d** and *ortho*-sulphonamide analogous **9a**–**b** displayed moderate submiclomolar *h*CA I inhibitory activity with *K*_I_s spanning in the range 105.3–864.4 nM.

It is worth stressing that shifting the primary sulphonamide functionality from the *ortho*-position (**9a**–**d**; *K*_I_s: 558.3–1562.0 nM) to the *meta*-position (**11a**–**d**; *K*_I_s: 105.3–663.1 nM), as well as shifting from the *meta*- to *para*-position (**13a**–**c**; *K*_I_s: 55.4–92.1 nM) dramatically enhanced the inhibitory activity towards *h*CA I. In addition, incorporation of the hydrazide linker in compound **16** (*K*_I_ = 81.4 nM) slightly improved *h*CA I inhibitory activity in comparison to its analogue **13b** (*K*_I_ = 92.1 nM). In contrast, grafting a 4-methyl group within the *meta*-sulphonamide derivatives **11a**–**c** led to compounds **11e**–**g** with much lower inhibitory activity (*K*_I_s: 2.256–4.521 μM, respectively).

The in vitro kinetic data demonstrated in Table 1 revealed that the physiologically dominant off-target *h*CA II isoform has been inhibited by the herein prepared **QBS** (**9a**–**d**, **11a**–**g**, **13a**–**c** and **16**) with *K*_I_s in the nanomolar range (from 7.3 to 998.2 nM), except compounds **11e** and **11g**, which displayed inhibitory activity in the low micromolar concentration (*K*_I_s = 1.503 and 2.356 μM, respectively). The *para*-sulphonamide derivative **13c** emerged as the most efficient *h*CA II inhibitor, in this study, with a single-digit nanomolar inhibition constant (*K*_I_ = 7.3 nM). Additionally, compounds **9a**–**c**, **13a**, **13b** and **16** exerted potent activities with *K*_I_ values equal 78.4, 49.7, 86.8, 36.5, 58.4 and 31.1 nM, respectively. 

It is noteworthy that switching the sulphonamide functionality from the *ortho*-position (**9a**–**d**; *K*_I_s: 49.7–112.4 nM) to the *meta*-position (**11a**–**d**; *K*_I_s: 154.8–365.7 nM) elicited a worsening of effectiveness toward the *h*CA II isoform, whereas shifting to the *para*-position led to compounds **13a**–**c** with an enhanced *h*CA II inhibitory activity. Moreover, incorporation of the 4-methyl group in compounds **11e**–**g** resulted in a decrease in the inhibitory activity (*K*_I_s: 998.2 nM–2.356 μM) in comparison to their analogues **11a**–**c** (*K*_I_s: 154.8 nM–365.7 nM), whereas the linker elongation approach (compound **16**) improved the inhibitory activity from 58.4 nM (for anilino derivative **13b**) to 31.1 nM, Table 1. 

The examined quinoline-based sulfonamides displayed potent to moderate inhibitory activity towards the target tumor-associated *h*CA IX isoform (*K*_I_ values spanning between 5.5 and 116.2 nM, Table 1), except compound **11f** (*K*_I_ = 853.4 nM). In particular, **QBS 11c** and **13b** emerged as excellent single-digit nanomolar *h*CA IX inhibitors (*K*_I_s = 8.4 and 5.5 nM, respectively). Additionally, sulfonamides **9d**, **13a**, **13c** and **16** exerted better or equipotent inhibitory activity (*K*_I_s = 25.9, 25.8, 18.6 and 21.7 nM, respectively) compared to the reference **AAZ** (*K*_I_ = 25 nM). 

Further analysis of the obtained results showed that grafting the sulfamoyl group at the *para*-position (**13a**–**c**; *K*_I_s: 5.5–25.8 nM) was more beneficial for *h*CA IX inhibitory activity than the *ortho*-substitution (**9a**–**d**; *K*_I_s: 25.9–65.6 nM) and the *meta*-substitution (**11a**–**d**; *K*_I_s: 8.4–86.5 nM), except for 6-chloro bearing derivative **11b**. Dissimilar to the inhibitory profile of target sulfonamides toward *h*CA I and II isoforms, the linker elongation approach failed to improve the *h*CA IX inhibitory activity (anilino derivative **13b**; *K*_I_ = 5.5 nM vs. hydrazido derivative **16**; *K*_I_ = 21.7 nM). It is worth noting that, appending a methyl group at C4 within the benzenesulfonamide moiety of **QBS 11a**–**c** resulted in **QBS 11e**–**g** analogues with about a 1.5- to 13.8-fold decreased potency, Table 1.

As shown in Table 1, the second cancer-related isoform here examined *h*CA XII has been potently inhibited by all the newly prepared quinoline-based sulfonamides **9a**–**d**, **11a**–**g**, **13a**–**c** and **16** (*K*_I_s range: 8.7–88.12 nM), except compound **11f**, which moderately affected the *h*CA XII isoform (*K*_I_ = 152.2 nM). Interestingly, grafting the zinc anchoring sulfamoyl group at the *para*-position achieved the best *h*CA XII inhibitory action in this study (**QBS 13a**–**c**; *K*_I_s = 9.8, 13.2 and 8.7 nM, respectively). Moreover, *ortho*-sulphonamide derivatives **9a** and **9d**, as well as the hydrazido derivative **16** possessed efficient inhibitory activity against the *h*CA XII isoform (*K*_I_s = 22.8, 26.5 and 25.4 nM, respectively). 

In conclusion, for both cancer-related isoforms *h*CA IX and *h*CA XII, grafting the sulfamoyl functionality at the *para*-position was more advantageous for inhibitory activity than the *ortho*-position, which, in turn, was more advantageous than *meta*-substitution. Furthermore, C4 substitution of the benzenesulfonamide moiety by a methyl group, as well as the incorporation of the hydrazide linker, slightly decreased the inhibitory activities toward both isoforms. 

The selectivity index (SI) presented in Table 2 obviously displayed a good selectivity profile for target **QBS** toward *h*CA IX over *h*CA I with **9d**, **11c**, **11e** and **11g** having the highest SIs (60.3, 52.7, 33.8 and 38.9, respectively). In addition, the **QBS** showed good selectivity toward *h*CA IX over *h*CA II, with **11c**, **11e**, **11g** and **13b** exhibiting the best SIs (18.4, 14.4, 20.3 and 10.6, respectively). Similarly, excellent selectivity toward *h*CA XII over *h*CA I was demonstrated by all the QBS except **11b**, **11d** and **16**; also, QBS showed a good selectivity towards *h*CA XII over *h*CA II with **11e** and **11g** being the highest (SIs: 17.1 and 25.4, respectively).

#### 2.2.2. Anticancer Activity

##### In Vitro Anti-Proliferative Activity

The CA inhibition data presented in Table 1 revealed that not only was an efficient single-digit nanomolar inhibition of CA IX isoform exerted by **QBS 11c** and **13b** (*K*_I_s = 8.4 and 5.5 nM, respectively), but also, both compounds demonstrated good selectivity toward the *h*CA IX isoform over the off-target isoforms *h*CA I (S.I. = 52.7 and 16.7, respectively) and *h*CA II (S.I. = 18.4 and 10.6, respectively), Table 2. Therefore, **QBS 11c** and **13b** were further screened for their potential in vitro anti-proliferative action against two breast cancer cell lines (MDA-MB-231 and MCF-7) under hypoxic conditions, exploiting a 72 h MTT assay protocol [31] and using Doxorubicin as a positive control drug. The IC_50_ values for the examined derivatives are listed in Table 3.

The results of the MTT assay are ascribed to both **QBS 11c** and **13b** potent anti-proliferative action toward the tested MDA-MB-231 and MCF-7 cell lines (IC_50_ range: from 0.43 ± 0.02 to 3.69 ± 0.17). Interestingly, **QBS 11c** showed submicro-molar activity against the MCF-7 cancer cell line (IC_50_ = 0.43 ± 0.02 μM). It is worth noting that **QBS 11c** displayed slight better activity (IC_50_ = 1.03 ± 0.05 μM and 0.43 ± 0.02 μM) than **QBS 13b** (IC_50_ = 2.24 ± 0.1 μM and 3.69 ± 0.17 μM) against MDA-MB-231 and MCF-7 cell lines, respectively (Table 3).

##### Effect of **QBS 11c** and **13b** on Apoptotic Markers Bax, Bcl-2, and Active Caspase-3

The levels of two members of Bcl-2 family proteins, the anti-apoptotic Bcl-2 protein, and the counteracting pro-apoptotic Bax protein, as well as the level of active caspase-3 (a key executioner protease) in MDA-MB-231 and MCF-7 cells have been assessed after incubation with **QBS 11c** and **13b** for 24 h. The obtained results (Table 4 and Table 5) revealed that the expression levels of the examined proteins (Bax, Bcl-2, and active caspase-3) have been significantly affected upon treatment with **QBS 11c** and **13b**.

Regarding MDA-MB-231 cells, their treatment with **QBS 11c** and **13b** resulted in significant elevation in the expression levels for Bax protein (by 7.1-fold and 6.3-fold, respectively) and active caspase-3 (by 4.93-fold and 3.62-fold, respectively), whereas such treatment led to a decrease in the expression levels for the anti-apoptotic Bcl-2 protein (by 55 and 63%, respectively) in comparison to the untreated control (Table 4).

On the other hand, treatment of MCF-7 cells with **QBS 11c** and **13b** led to an increase in the expression levels for Bax protein (by 5.36-fold and 4.64-fold, respectively) and active caspase-3 (by 3.64-fold and 3.02-fold, respectively); in addition, it resulted in a suppression of the expression levels for Bcl-2 protein (by 21 and 38%, respectively) compared to the untreated control (Table 5).

### 2.3. Molecular Modeling Studies

Molecular docking and MM-GBSA-based refinements inside *h*CA isozymes IX and XII (PDB 5FL4 [32] and 4WW8 [33]) were employed to explore the binding modalities of the synthesized compounds and to correlate their structural characteristics with the inhibition activity. The co-crystalized ligands in both *h*CA isoforms IX and XII showed the usual pattern of the sulfonamide moiety where it binds to the zinc(II) ion after the displacement of the metal-bound water molecule to form the tetrahedral adduct with the zinc atom. Both ligands are positioned toward the hydrophobic half of the active site and the ligand binding is achieved mostly by van der Waals and hydrophobic interactions [32,33]. 

**Figure 3:**
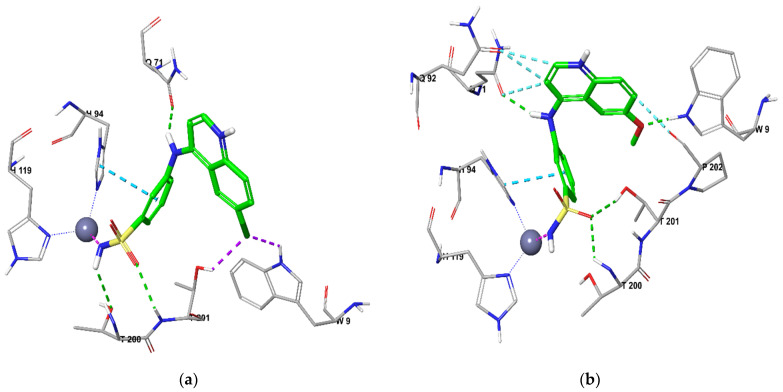
Docking CA IX for (**a**) **11c** and (**b**) **13b**.

The benzenesulfonamide ring fit deeply inside the shallow CA active site for both isoforms anchoring the zinc atom in a typical manner for sulfonamide CAIs through an NH^−^---Zn^2+^ bond. In addition, in the active site of CA IX, two hydrogen bonds occurred between the sulfamoyl NH---O (T200) and sulfamoyl S = O---HN (T201) for **11c**, while **13b** sulfamoyl S = O made two hydrogen bonds with T200 (NH) and T201(OH) alongside the π−π stacking between H94 and the sulfonamide benzene ring in both **11c** and **13b** (Figure 3).Furthermore, **11c** was a correct distance from the additional three hydrogen bonds by its linker NH with Q71 (C = O) and by its halogen that made two interactions with W9 (NH) and T201 (OH). On the other hand, **13b** had only two additional hydrogen bonds (linker NH---C = O of Q71 and CH_3_O---NH of W9) in concurrence with the hydrophobic interaction of its quinoline ring with Q71, Q92 and P202.

**Figure 4:**
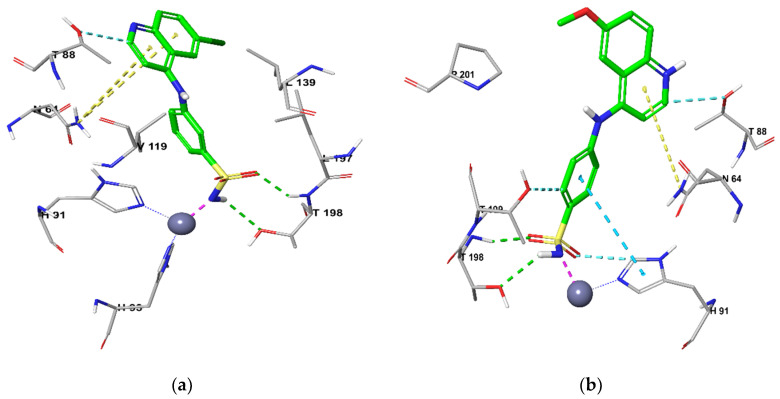
Docking CA XII for (**a**) **11c** and (**b**) **13b**.

Similarly, docking of **QBS** into CAXII revealed two hydrogen bonds (sulfamoyl NH---O (T198) and sulfamoyl S = O---HN (T198) for both **11b** and **13c** (Figure 4). Moreover, the quinoline ring in both **QBS** engaged in a N-π interaction with N64 and hydrophobic interaction with T88. Additionally, the sulfonamide benzene ring in **13b** was observed to make a π−π stacking interaction with H94.

The target **QBS** showed a similar interaction to the co-crystalized ligand maintaining the sulfonamide moiety interactions; in addition, the quinoline ring was similarly positioned toward the hydrophobic half of the active site of *h*CA IX and XII, similar to the co-crystalized ligands interaction.

The narrow range of docking scores and prime MMGBSA binding energy was in accordance with the observed in vitro inhibitory activity of **11c** and **13b** on each isozyme, as shown in Table 6.

## 3. Materials and Methods

### 3.1. Chemistry

***General***

All reaction solvents and reagents were purchased from commercial suppliers; Sigma-Aldrich (Sigma-Aldrich Chemie GmbH, Taufkirchen, Germany) and Alfa Aesar(Thermo Fisher GmbH, Kandel, Germany) and used without further purification. Melting points were measured with a Stuart melting point apparatus and were uncorrected. The NMR spectra were obtained on Bruker Avance 400 (400 MHz ^1^H and 100 MHz ^13^C NMR). ^1^H NMR spectra were referenced to tetramethylsilane (*δ* = 0.00 ppm) as an internal standard and were reported as follows: chemical shift, multiplicity (b = broad, s = singlet, d = doublet, t = triplet, dd = doublet of doublet, m = multiplet). IR spectra were recorded with a Bruker FT-IR spectrophotometer. Reaction courses and product mixtures were routinely monitored using thin layer chromatography (TLC) that was carried out using aluminum sheets pre-coated with silica gel 60 F_254_ purchased from Merk (Merck Group, Darmstadt, Germany) Compounds **5**–**7** and **15** were prepared according to the reported methods [27,34,35].

#### General Procedures for Preparation of Target **QBS** (**9a**–**d**, **11a**–**g**, **13a**–**c** and **16**)

To a heated solution of the appropriate 4-chloroquinoline derivative **7a**–**d** (0.5 mmol) in dry isopropanol (3 mL) in a round-bottom flask, a catalytic amount of HCl, then the appropriate benzenesulfonamide derivatives **8**, **10a**–**b**, **12** and **15** (0.5 mmol) were added. The reaction mixture was stirred under reflux temperature for 2 h. The precipitated solid was collected by filtration while hot, washed with cold water, cold ethanol and petroleum ether then recrystallized from methanol to produce the target **QBS** derivatives **9a**–**d**, **11a**–**g**, **13a**–**c** and **16**.

Synthesis of 2-((6-methylquinolin-4-yl)amino)benzenesulfonamide (**9a**). **QBS 9a** was obtained following the general procedure mentioned above using **7a** (0.09 g, 0.50 mmol) and 2-aminobenzenesulfonamide **8** (0.086 g, 0.50 mmol). A 78% yield; m.p. 264–265 °C; IR (KBr, ν cm^−1^): 3369, 3249, 3146 (NH, NH_2_), and 1338, 1163 (SO_2_); ^1^H NMR (DMSO-*d*_6_) *δ ppm*: 2.58 (s, 3H, CH_3_), 6.2 (d, 1H, Ar-H, H3 of quinoline, *J* = 6.4 Hz), 7.62 (d, 1H, Ar-H, H3 of C_6_H_4_SO_2_NH_2_, *J* = 7.6 Hz), 7.73 (t, 1H, Ar-H, H5 of C_6_H_4_SO_2_NH_2_, *J* = 7.6 Hz), 7.78 (s, 2H, NH_2_, D_2_O exchangeable of -SO_2_NH_2_), 7.84 (t, 1H, Ar-H, H4 of C_6_H_4_SO_2_NH_2_, *J* = 7.6, Hz), 7.89 (d, 1H, Ar-H, H7 of quinoline, *J* = 8.8 Hz), 8.02 (d, 1H, Ar-H, H8 of quinoline, *J* = 8.8 Hz), 8.11 (d, 1H, Ar-H, H6 of C_6_H_4_SO_2_NH_2_, *J* = 7.6 Hz), 8.43 (d, 1H, Ar-H, H2 of quinoline, *J* = 6.4 Hz), 8.98 (s, 1H, Ar-H, H5 of quinoline), 11.00 (s, 1H, NH, D_2_O exchangeable); ^13^C NMR (DMSO-*d*_6_) *δ ppm*: 21.60, 101.02, 117.60, 120.23, 123.80, 129.58, 129.69, 131.11, 134.46, 134.69, 135.98, 136.81, 137.40, 141.44, 142.01, 156.46; Anal. Calcd. for C_16_H_15_N_3_O_2_S: C, 61.32; H, 4.82; N, 13.41; found C, 61.44; H, 4.85; N, 13.32.

Synthesis of 2-((6-methoxyquinolin-4-yl)amino)benzenesulfonamide (**9b**). **QBS 9b** was obtained following the general procedure mentioned above using **7b** (0.1 g, 0.50 mmol) and 2-aminobenzenesulfonamide **8** (0.086 g, 0.50 mmol). A 65% yield; m.p. 275–277 °C; IR (KBr, ν cm^−1^): 3457, 3218, 3124 (NH, NH_2_) and 1341, 1167 (SO_2_); ^1^H NMR (DMSO-*d*_6_) *δ ppm*: 4.03 (s, 3H, -OCH_3_), 6.18 (d, 1H, Ar-H, H3 of quinoline, *J* = 6.8 Hz), 7.63 (d, 1H, Ar-H, H3 of C_6_H_4_SO_2_NH_2_, *J* = 7.6 Hz), 7.66–7.75 (m, 2H, Ar-H, H7 of quinoline and H5 of C_6_H_4_SO_2_NH_2_), 7.76 (s, 2H, NH_2_, D_2_O exchangeable of -SO_2_NH_2_), 7.85 (t, 1H, Ar-H, H4 of C_6_H_4_SO_2_NH_2_, *J* = 7.0 Hz), 8.02 (d, 1H, Ar-H, H8 of quinoline *J* = 8.8 Hz), 8.11 (d, 1H, Ar-H, H6 of C_6_H_4_SO_2_NH_2_, *J* = 7.0 Hz), 8.38 (s, 1H, Ar-H, H5 of quinoline), 8.49 (d, 1H, Ar-H, H2 of quinoline *J* = 6.8 Hz), 11.01 (s, 1H, NH, D_2_O exchangeable); ^13^C NMR (DMSO-*d*_6_) *δ ppm*: 57.26, 101.03, 104.21, 118.90, 122.18, 125.90, 129.57, 129.70, 131.32, 133.72, 134.49, 134.84, 140.72, 141.65, 156.10, 158.43; Anal. Calcd. for C_16_H_15_N_3_O_3_S: C, 58.35; H, 4.59; N, 12.76; found C, 58.67; H, 4.62; N, 12.68.

Synthesis of 2-((6-chloroquinolin-4-yl)amino)benzenesulfonamide (**9c**). **QBS 9c** was obtained following the general procedure mentioned above using **7c** (0.1 g, 0.50 mmol) and 2-aminobenzenesulfonamide **8** (0.086 g, 0.50 mmol). A 71% yield; m.p. 258–260 °C; IR (KBr, ν cm^−1^): 3257, 3144, 3102 (NH, NH_2_) and 1335, 1163 (SO_2_); ^1^H NMR (DMSO-*d*_6_) *δ ppm*: 6.22 (d, 1H, Ar-H, H3 of quinoline, *J* = 6.8 Hz), 7.63 (d, 1H, Ar-H, H3 of C_6_H_4_SO_2_NH_2_, *J* = 7.6 Hz), 7.68–7.82 (m, 3H, Ar-H, H5 of C_6_H_4_SO_2_NH_2_, NH_2_, D_2_O exchangeable of -SO_2_NH_2_), 7.85 (t, 1H, Ar-H, H4 of C_6_H_4_SO_2_NH_2_, *J* = 6.8, 7.6 Hz), 8.02–8.19 (m, 3H, Ar-H, H2, H7 and H8 of quinoline), 8.5 (d, 1H, Ar-H, H6 of C_6_H_4_SO_2_NH_2_, *J* = 7.2 Hz), 9.31 (s, 1H, Ar-H, H5 of quinoline), 11.01 (s, 1H, NH, D_2_O exchangeable); ^13^C NMR (DMSO-*d*_6_) *δ ppm*: 101.84, 118.59, 122.67, 124.38, 129.59, 129.98, 131.15, 131.99, 134.35, 134.57, 134.57, 137.29, 141.57, 143.01, 156.39; Anal. Calcd. for C_15_H_12_ClN_3_O_2_S: C, 53.98; H, 3.62; N, 12.59; found C, 54.21; H, 3.58; N, 12.51.

Synthesis of 2-((5,7-bis(trifluoromethyl)quinolin-4-yl)amino)benzenesulfonamide (**9d**). **QBS 9d** was obtained following the general procedure mentioned above using **7**d (0.15 g, 0.50 mmol) and 2-aminobenzenesulfonamide **8** (0.086 g, 0.50 mmol). A 78% yield; m.p. 240–242 °C; IR (KBr, ν cm^−1^): 3331, 3159, 3115 (NH, NH_2_), 1583 (C = N) and 1342, 1165 (SO_2_); ^1^H NMR (DMSO-*d*_6_) *δ ppm*: 6.84 (t, 1H, Ar-H, H5 of C_6_H_4_SO_2_NH_2_, *J* = 7.2, 7.6 Hz ), 7.01 (d, 1H, Ar-H, H3 of quinoline, *J* = 8.0 Hz), 7.33–7.47 (m, 7H, Ar-H, NH_2_, D_2_O exchangeable of -SO_2_NH_2_), 7.63 (d, 1H, Ar-H, H2 of quinoline, *J* = 8.0 Hz); Anal. Calcd. for C_17_H_11_F_6_N_3_O_2_S: C, 46.90; H, 2.55; N, 9.65; found C, 47.05; H, 2.53; N, 9.73.

Synthesis of 3-((6-methylquinolin-4-yl)amino)benzenesulfonamide (**11a**). **QBS 11a** was obtained following the general procedure mentioned above using **7a** (0.09 g, 0.50 mmol) and 3-aminobenzenesulfonamide **10a** (0.086 g, 0.50 mmol). A 75% yield; m.p. 272–274 °C; IR (KBr, ν cm^−1^): 3286, 3148, 3107 (NH, NH_2_) and 1348, 1156 (SO_2_); ^1^H NMR (DMSO-*d*_6_) *δ ppm*: 2.58 (s, 3H, CH_3_), 6.91 (d, 1H, Ar-H, H3 of quinoline, *J* = 4.8 Hz), 7.61 (s, 2H, NH_2_, D_2_O exchangeable of -SO_2_NH_2_), 7.77 (d, 2H, Ar-H, H4 and H6 of C_6_H_4_SO_2_NH_2_, *J* = 8.4 Hz), 7.84–7.96 (m, 3H, Ar-H, H7 of quinoline, H3 and H5 of C_6_H_4_SO_2_NH_2_), 8.08 (d, 1H, Ar-H, H8 of quinoline, *J* = 8.4, Hz), 8.55 (d, 1H, Ar-H, H2 of quinoline, *J* = 6.8 Hz), 8.74 (s, 1H, Ar-H, H5 of quinoline), 11.14 (s, 1H, NH, D_2_O exchangeable); ^13^C NMR (DMSO-*d*_6_) *δ ppm*: 21.69, 100.27, 117.87, 120.50, 122.51, 123.20, 124.51, 128.79, 131.18, 136.11, 137.01, 137.77, 138.42, 142.67, 146.11, 154.49; Anal. Calcd. for C_16_H_15_N_3_O_2_S: C, 61.32; H, 4.82; N, 13.41; found C, 61.07; H, 4.86; N, 13.33.

Synthesis of 3-((6-methoxyquinolin-4-yl)amino)benzenesulfonamide (**11b**). **QBS 11b** was obtained following the general procedure mentioned above using **7b** (0.1 g, 0.50 mmol) and 3-aminobenzenesulfonamide **10a** (0.086 g, 0.50 mmol). A 69% yield; m.p. 244–246 °C; IR (KBr, ν cm^−1^): 3349, 3152, 3111 (NH, NH_2_) and 1341, 1155 (SO_2_); ^1^H NMR (DMSO-*d*_6_) *δ ppm*: 3.99 (s, 3H,-OCH_3_), 6.90 (d, 1H, Ar-H, H3 of quinoline, *J* = 6.8 Hz), 7.60 (s, 2H, NH_2_, D_2_O exchangeable of -SO_2_NH_2_), 7.69 (d, 1H, Ar-H, H7 of quinoline, *J* = 9.2 Hz), 7.76–7.88 (m, 3H, Ar-H, H4, H5 and H6 of C_6_H_4_SO_2_NH_2_,), 7.94 (s, 1H, Ar-H, H3 of C_6_H_4_SO_2_NH_2_), 8.11 (d, 1H, Ar-H, H8 of quinoline, *J* = 9.2 Hz), 8.33 (s, 1H, Ar-H, H5 of quinoline), 8.50 (d, 1H, Ar-H, H2 of quinoline, *J* = 6.8 Hz), 11.18 (s, 1H, NH, D_2_O exchangeable); ^13^C NMR (DMSO-*d*_6_) *δ ppm*: 57.11, 100.22, 103.48, 119.20, 122.42, 122.60, 124.43, 126.10, 128.95, 131.18, 133.97, 138.55, 141.22, 146.11, 153.97, 158.60; Anal. Calcd. for C_16_H_15_N_3_O_3_S: C, 58.35; H, 4.59; N, 12.76; found C, 58.59; H, 4.63; N, 12.70.

Synthesis of 3-((6-chloroquinolin-4-yl)amino)benzenesulfonamide (**11c**). **QBS 11c** was obtained following the general procedure mentioned above using **7c** (0.1 g, 0.50 mmol) and 3-aminobenzenesulfonamide **10a** (0.086 g, 0.50 mmol). A 70% yield; m.p. 272–274 °C; IR (KBr, ν cm^−1^): 3291, 3159, 3100 (NH, NH_2_) and 1348, 1156 (SO_2_); ^1^H NMR (DMSO-*d*_6_) *δ ppm*: 6.96 (d, 1H, Ar-H, H3 of quinoline, *J* = 6.8 Hz), 7.63 (s, 2H, NH_2_, D_2_O exchangeable of -SO_2_NH_2_) 7.74–7.81 (m, 2H, Ar-H, H4 and H5 of C_6_H_4_SO_2_NH_2_), 7.87 (d, 1H, Ar-H, H6 of C_6_H_4_SO_2_NH_2_, *J* = 4.4, Hz), 7.93 (s, 1H, Ar-H, H3 of C_6_H_4_SO_2_NH_2_), 8.10 (d, 1H, Ar-H, H7 of quinoline, *J* = 9.2 Hz), 8.23 (d, 1H, Ar-H, H8 of quinoline, *J* = 9.2 Hz), 8.61 (d, 1H, Ar-H, H2 of quinoline, *J* = 6.8 Hz), 9.12 (s, 1H, Ar-H, H5 of quinoline), 11.37 (s, 1H, NH, D_2_O exchangeable);^13^C NMR (DMSO-*d*_6_) *δ ppm*: 101.00, 118.92, 122.54, 122.91, 123.79, 124.79, 128.79, 130.82, 131.27, 134.66, 137.50, 138.08, 143.71, 146.15, 154.39; Anal. Calcd. for C_15_H_12_ClN_3_O_2_S: C, 53.98; H, 3.62; N, 12.59; found C, 54.20; H, 3.59; N, 12.66.

Synthesis of 3-((5,7-bis(trifluoromethyl)quinolin-4-yl)amino)benzenesulfonamide (**11d**). **QBS 11d** was obtained following the general procedure mentioned above using **7d** (0.15 g, 0.50 mmol) and 3-aminobenzenesulfonamide **10a** (0.086 g, 0.50 mmol). A 78% yield; m.p. 172–174 °C; IR (KBr, ν cm^−1^): 3331, 3159, 3115 (NH, NH_2_), 1583 (C = N) and 1342, 1165 (SO_2_); ^1^H NMR (DMSO-*d*_6_) *δ ppm*: 7.49 (d, 2H, Ar-H, H4 and H6 of C_6_H_4_SO_2_NH_2_, *J* = 7.6 Hz), 7.50 - 7.68 (m, 7H, Ar-H, NH_2_, D_2_O exchangeable of -SO_2_NH_2_), 7.71 (s, 1H, Ar-H, H6 of quinoline), 7.73 (s, 1H, NH, D_2_O exchangeable); Anal. Calcd. for C_17_H_11_F_6_N_3_O_2_S: C, 46.90; H, 2.55; N, 9.65; found C, 46.69; H, 2.60; N, 9.57.

Synthesis of 2-methyl-5-((6-methylquinolin-4-yl)amino)benzenesulfonamide (**11e**). **QBS 11e** was obtained following the general procedure mentioned above using **7a** (0.09 g, 0.50 mmol) and 5-amino-2-methylbenzenesulfonamide **10b** (0.093 g, 0.50 mmol). A 66% yield; m.p. 280–282 °C; IR (KBr, ν cm^−1^): 3324, 3205, 3144 (NH, NH_2_) and 1333, 1163 (SO_2_); ^1^H NMR (DMSO-*d*_6_) *δ ppm*: 2.57 (s, 3H, CH_3_ of quinoline), 2.66 (s, 3H, CH_3_ of C_6_H_4_SO_2_NH_2_), 6.84 (d, 1H, Ar-H, H3 of quinoline, *J* = 6.8 Hz), 7.52–7.69 (m, 4H, Ar-H, H3 and H4 of C_6_H_4_SO_2_NH_2_, NH_2_, D_2_O exchangeable of -SO_2_NH_2_), 7.88 (d, 1H, Ar-H, H7 of quinoline, *J* = 8.8, Hz), 7.94 (s, 1H, Ar-H, H6 of C_6_H_4_SO_2_NH_2_), 8.06(d, 1H, Ar-H, H8 of quinoline, *J* = 8.8, Hz), 8.50 (d, 1H, Ar-H, H2 of quinoline, *J* = 6.8, Hz), 8.73 (s, 1H, Ar-H H5 of quinoline), 11.07 (s, 1H, NH, D_2_O exchangeable); ^13^C NMR (DMSO-*d*_6_) *δ ppm*: 19.94, 21.67, 100.12, 117.73, 120.47, 123.18, 124.25, 128.89, 134.20, 135.20, 135.73, 136.03, 136.98, 137.66, 142.51, 143.80, 154.70; Anal. Calcd. for C_17_H_17_N_3_O_2_S: C, 62.37; H, 5.23; N, 12.83; found C, 62.54; H, 5.26; N, 12.91.

Synthesis of 5-((6-methoxyquinolin-4-yl)amino)-2-methylbenzenesulfonamide (**11f**). **QBS 11f** was obtained following the general procedure mentioned above using **7b** (0.1 g, 0.50 mmol) and 5-amino-2-methylbenzenesulfonamide **10b** (0.093 g, 0.50 mmol). A 68% yield; m.p. 248–250 °C; IR (KBr, ν cm^−1^): 3378, 3203, 3146 (NH, NH_2_) and 1331, 1155 (SO_2_); ^1^H NMR (DMSO-*d*_6_) *δ ppm*: 2.67 (s, 3H, CH_3_), 4.01 (s, 3H, -OCH_3_), 6.84 (d, 1H, Ar-H, H3 of quinoline, *J* = 6.8 Hz), 7.49–7.74 (m, 5H, Ar-H, H7 of quinoline, H3 and H4 of C_6_H_4_SO_2_NH_2_,NH_2_, D_2_O exchangeable of -SO_2_NH_2_), 7.94 (s, 1H, Ar-H, H6 of C_6_H_4_SO_2_NH_2_), 8.09 (d, 1H, Ar-H, H8 of quinoline, *J* = 9.2, Hz), 8.31 (s, 1H, Ar-H, H5 of quinoline), 8.45 (d, 1H, Ar-H, H2 of quinoline, *J* = 6.8, Hz), 11.10 (s, 1H, NH, D_2_O exchangeable); ^13^C NMR (DMSO-*d*_6_) *δ ppm*: 19.94, 57.09, 100.05, 103.51, 119.05, 122.38, 124.35, 125.98, 129.08, 133.91, 134.22, 135.14, 135.83, 141.09, 143.80, 154.21, 158.55; Anal. Calcd. for C_17_H_17_N_3_O_3_S: C, 59.46; H, 4.99; N, 12.24; found C, 59.23; H, 5.03; N, 12.32.

Synthesis of 5-((6-chloroquinolin-4-yl)amino)-2-methylbenzenesulfonamide (**11g**). **QBS 11g** was obtained following the general procedure mentioned above using **7c** (0.1 g, 0.50 mmol) and 5-amino-2-methylbenzenesulfonamide **10b** (0.093 g, 0.50 mmol). A 77% yield; m.p. 282–285 °C; IR (KBr, ν cm^−1^): 3277, 3204, 3174 (NH, NH_2_) and 1326, 1156 (SO_2_); ^1^H NMR (DMSO-*d*_6_) *δ ppm*: 2.66 (s, 3H, CH_3_), 6.89 (d, 1H, Ar-H, H3 of quinoline, *J* = 6.8 Hz), 7.54–7.69 (m, 4H, Ar-H, H3 and H4 of C_6_H_4_SO_2_NH_2_, NH_2_, D_2_O exchangeable of -SO_2_NH_2_), 7.94 (s, 1H, Ar-H, H6 of C_6_H_4_SO_2_NH_2_), 8.08 (d, 1H, Ar-H, H7 of quinoline, *J* = 9.2, Hz), 8.20 (d, 1H, Ar-H, H8 of quinoline, *J* = 9.2, Hz), 8.57 (d, 1H, Ar-H, H2 of quinoline, *J* = 6.8, Hz), 9.09 (s, 1H, Ar-H, H5 of quinoline), 11.28 (s, 1H, NH, D_2_O exchangeable); ^13^C NMR (DMSO-*d*_6_) *δ ppm*: 19.94, 100.87, 118.81, 122.94, 123.69, 124.25, 128.83, 132.22, 134.28, 134.59, 135.40, 135.51, 137.51, 143.61, 143.85, 154.56; Anal. Calcd. for C_16_H_14_ClN_3_O_2_S: C, 55.25; H, 4.06; N, 12.08; found C, 54.96; H, 4.08; N, 11.99.

Synthesis of 4-((6-methylquinolin-4-yl)amino)benzenesulfonamide (**13a**). **QBS 13a** was obtained following the general procedure mentioned above using **7a** (0.09 g, 0.50 mmol) and 4-aminobenzenesulfonamide **12** (0.086 g, 0.50 mmol). A 70% yield; m.p. 270–272°C; IR (KBr, ν cm^−1^): 3335, 3216, 3159 (NH, NH_2_) and 1324, 1156 (SO_2_); ^1^H NMR (DMSO-*d*_6_) *δ ppm*: 2.57 (s, 3H, CH_3_), 7.01 (d, 1H, Ar-H, H3 of quinoline, *J* = 6.8 Hz), 7.53 (s, 2H, NH_2_, D_2_O exchangeable of -SO_2_NH_2_), 7.73 (d, 2H, Ar-H, H3 and H5 of C_6_H_4_SO_2_NH_2_, *J* = 8.4, Hz), 7.89 (d, 1H, Ar-H, H7 of quinoline, *J* = 8.8, Hz), 7.99 (d, 2H, Ar-H, H2 and H6 of C_6_H_4_SO_2_NH_2_, *J* = 8.4, Hz), 8.07 (d, 1H, Ar-H, H8 of quinoline, *J* = 8.8 Hz), 8.55 (d, 1H, Ar-H, H2 of quinoline, *J* = 6.8, Hz), 8.78 (s, 1H, Ar-H, H5 of quinoline), 11.18 (s, 1H, NH, D_2_O exchangeable); ^13^C NMR (DMSO-*d*_6_) *δ ppm*: 21.67, 100.80, 118.07, 120.50, 123.30, 125.33, 125.33, 127.85, 127.85, 136.14, 137.04, 137.82, 141.09, 142.42, 142.69, 154.23; Anal. Calcd. for C_16_H_15_N_3_O_2_S: C, 61.32; H, 4.82; N, 13.81; found C, 61.49; H, 4.86; N, 13.89.

Synthesis of 4-((6-methoxyquinolin-4-yl)amino)benzenesulfonamide (**13b**). **QBS 13b** was obtained following the general procedure mentioned above using **7b** (0.1 g, 0.50 mmol) and 4-aminobenzenesulfonamide **12** (0.086 g, 0.50 mmol). A 67% yield; m.p. 250–252 °C; IR (KBr, ν cm^−1^): 3333, 3192, 3107 (NH, NH_2_) and 1338, 1162 (SO_2_); ^1^H NMR (DMSO-*d*_6_) *δ ppm*: 4.03 (s, 3H, -OCH_3_), 7.01 (d, 1H, Ar-H, H3 of quinoline, *J* = 6.8 Hz), 7.53 (s, 2H, NH_2_, D_2_O exchangeable of -SO_2_NH_2_), 7.69 (dd, 1H, Ar-H, H7 of quinoline, *J* = 9.2 Hz), 7.75 (d, 2H, Ar-H, H3 and H5 of C_6_H_4_SO_2_NH_2_, *J* = 8.4, Hz), 7.99 (d, 2H, Ar-H, H2 and H6 of C_6_H_4_SO_2_NH_2_, *J* = 8.4, Hz), 8.10 (d, 1H, Ar-H, H8 of quinoline, *J* = 9.2 Hz), 8.37 (s, 1H, Ar-H, H5 of quinoline), 8.50 (d, 1H, Ar-H, H2 of quinoline, *J* = 6.8, Hz), 11.24 (s, 1H, NH, D_2_O exchangeable); ^13^C NMR (DMSO-*d*_6_) *δ ppm*: 57.18, 100.77, 103.63, 119.44, 122.41, 125.44, 125.44, 126.16, 127.82, 127.82, 134.01, 141.21, 141.23, 142.34, 153.70, 158.61; Anal. Calcd. for C_16_H_15_N_3_O_3_S: C, 58.35; H, 4.59; N, 12.76; found C, 58.61; H, 4.55; N, 12.83.

Synthesis of 4-((6-chloroquinolin-4-yl)amino)benzenesulfonamide (**13c**). **QBS 13c** was obtained following the general procedure mentioned above using **7c** (0.1 g, 0.50 mmol) and 4-aminobenzenesulfonamide **12** (0.086 g, 0.50 mmol). A 72% yield; m.p. 284–286 °C; IR (KBr, ν cm^−1^): 3281, 3208, 3148 (NH, NH_2_) and 1330, 1166 (SO_2_); ^1^H NMR (DMSO-*d*_6_) *δ ppm*: 7.04 (d, 1H, Ar-H, H3 of quinoline, *J* = 6.8 Hz), 7.54 (s, 2H, NH_2_, D_2_O exchangeable of -SO_2_NH_2_), 7.73 (d, 2H, Ar-H, H3 and H5 of C_6_H_4_SO_2_NH_2_, *J* = 8.0, Hz), 8.00 (d, 2H, Ar-H, H2 and H6 of C_6_H_4_SO_2_NH_2_, *J* = 8.0, Hz), 8.09 (d, 1H, Ar-H, H7 of quinoline, *J* = 8.8 Hz), 8.21 (d, 1H, Ar-H, H8 of quinoline, *J* = 8.8, Hz), 8.62 (d, 1H, Ar-H, H2 of quinoline, *J* = 6.8, Hz), 9.16 (s, 1H, Ar-H, H5 of quinoline), 11.18 (s, 1H, NH, D_2_O exchangeable); ^13^C NMR (DMSO-*d*_6_) *δ ppm*: 101.50, 119.07, 122.91, 123.85, 125.42, 125.42, 127.89, 127.89, 132.35, 134.67, 137.53, 140.73, 142.70, 143.73, 154.11; Anal. Calcd. for C_15_H_12_ClN_3_O_2_S: C, 53.98; H, 3.62; N, 12.59; found C, 54.17; H, 3.66; N, 12.53.

Synthesis of 4-(2-(6-Methoxyquinolin-4-yl)hydrazine-1-carbonyl)benzenesulfonamide (**16**). **QBS 16** was obtained following the general procedure mentioned above using **7b** (0.1 g, 0.50 mmol) and 4-(hydrazinecarbonyl)benzenesulfonamide **15** (0.108 g, 0.50 mmol). A 69% yield; m.p. 228–230 °C; IR (KBr, ν cm^−1^): 3345, 3208, 3119, 3066 (NH, NH_2_), 1685 (C = O) and 1333, 1162 (SO_2_); ^1^H NMR (DMSO-*d*_6_) *δ ppm*: 3.99 (s, 3H, -OCH_3_), 7.01 (d, 1H, Ar-H, H3 of quinoline, *J* = 6.8 Hz), 7.56 (s, 1H, Ar-H, H5 of quinoline), 7.63 (s, 2H, NH_2_, D_2_O exchangeable of -SO_2_NH_2_), 7.72 (dd, 1H, Ar-H, H7 of quinoline, *J* = 8.4, Hz), 7.94 (d, 1H, Ar-H, H3 of C_6_H_4_SO_2_NH_2_, *J* = 8.8, Hz), 8.01–8.11 (m, 3H, Ar-H, H2, H5 and H6 of C_6_H_4_SO_2_NH_2_,), 8.23 (d, 1H, Ar-H, H8 of quinoline, *J* = 8.4 Hz), 8.55 (d, 1H, Ar-H, H2 of quinoline, *J* = 6.8 Hz), 11.23 (s, 1H, NH, D_2_O exchangeable), 11.66 (s, 1H, NHC=O, D_2_O exchangeable); ^13^C NMR (DMSO-*d*_6_) *δ ppm*: 56.87, 98.96, 102.66, 116.73, 122.60, 126.28, 126.44, 128.54, 129.05, 133.52, 135.08, 147.25, 147.83, 155.92, 158.51, 165.27, 165.59; Anal. Calcd. for C_17_H_16_N_4_O_4_S: C, 54.83; H, 4.33; N, 15.05; found C, 55.06; H, 4.31; N, 14.96.

### 3.2. Biological Evaluation

All adopted procedures for the conducted in vitro biological assays were performed as described earlier; CA (stopped-flow [4,30,36]), cytotoxicity (MTT [37,38]), and assessment of apoptotic markers [39,40] assays, as well as induction of hypoxia with cobalt chloride [41,42], and were mentioned in the Supporting Materials.

### 3.3. Molecular Modelling

The utilized procedures within the docking experiments for **QBS 11c** and **13b** in *h*CA IX (pdb: 5FL4, [32]) and *h*CA XII (pdb: 4WW8, [33]) active sites are provided in the Supplementary Materials.

## 4. Conclusions

In this work, the design, synthesis and characterization of different novel series of quinoline-based sulfonamides (**QBS**; **9a**–**c**, **11a**–**h**, **13a**–**c** and **16**) were reported, afterwards their CA inhibition activity were examined against *h*CA I, II, IX and XII. Most of the newly reported **QBS** efficiently inhibited the herein investigated *h*CA IX and XII (tumor-related isoforms) with *K*_I_s in the ranges 5.5–853.4 nM and 8.7–152.2 nM, respectively. Furthermore, **QBS 9a**, **9b**, **9d**, **13a**, **13c** and **16** showed *K_I_*s values in the nanomolar range from 18.6–39.2 nM and compounds **11c** and **13b** were shown to be the most effective *h*CA IX inhibitor in this investigation (*K*_I_s = 8.4 and 5.5 nM, respectively). Similarly, **13a** and **13c** showed one-digit nanomolar inhibition activity against *h*CA XII (*K*_I_s = 9.8 and 8.7 nM, respectively). In addition, **11c** and **13b** showed good selectivity towards *h*CA IX over the physiological isomer *h*CAI (SI = 52.7 and 16.7) and *h*CAII (SI = 18.4 and 10.6). SAR analysis pointed out that grafting the sulfamoyl functionality at the *para*-position was more advantageous for *h*CA IX and *h*CA XII inhibitory activities than the *ortho*-position, which, in turn, was more advantageous than *meta*-substitution. Additionally, the C4 substitution of the benzenesulfonamide moiety by a methyl group, as well as the incorporation of the hydrazide linker, slightly reduced the inhibitory activities toward both the *h*CA IX and *h*CA XII isoforms. Thereafter, a utilized MTT assay revealed that **QBS 11c** (IC_50_ = 1.03 ± 0.05 μM and 0.43 ± 0.02 μM) and **QBS 13b** (IC_50_ = 2.24 ± 0.1 μM and 3.69 ± 0.17 μM) possessed potent activity against MDA-MB-231 and MCF-7 cell lines, respectively, under hypoxic conditions. Moreover, the incubation of MDA-MB-231 and MCF-7 cells with **QBS 11b** and **13b** enhanced the expression levels for pro-apoptotic markers Bax and active Caspase-3 proteins, while the level of anti-apoptotic Bcl-2 protein was suppressed. Finally, the molecular docking simulations have provided insights for the binding interactions of **QBS 11b** and **13b** within *h*CA IX (pdb: 5FL4) and *h*CA XII (pdb: 4WW8) binding sites.

## Figures and Tables

**Table 1:**
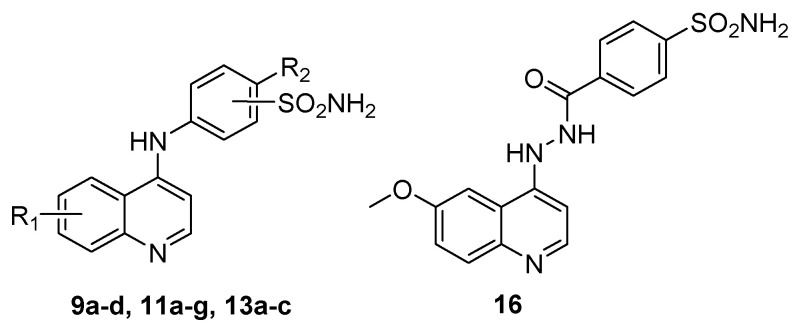
Inhibition data for the target sulfonamides on human CA isoforms *h*CA I, II, IX and XII using AAZ as a reference drug.

cmpd	*o-/m-/p-*	R_1_	R_2_	*K*_I_ (nM) ^1^			
				CA I	CA II	CA IX	CA XII
**9a**	*o-*	6-CH_3_	H	558.3	78.4	36.4	22.8
**9b**	*o-*	6-OCH_3_	H	864.4	49.7	39.2	42.1
**9c**	*o-*	6-Cl	H	1239	86.8	65.6	38.4
**9d**	*o-*	5,7-diCF_3_	H	1562	112.4	25.9	26.5
**11a**	*m-*	6-CH_3_	H	663.1	265.2	70.1	41.2
**11b**	*m-*	6-OCH_3_	H	258.4	365.7	86.5	73.1
**11c**	*m-*	6-Cl	H	442.3	154.8	8.4	55.3
**11d**	*m-*	5,7-diCF_3_	H	105.3	221.0	52.9	34.0
**11e**	*m-*	6-CH_3_	CH_3_	3498	1503	103.5	88.1
**11f**	*m-*	6-OCH_3_	CH_3_	2256	998.2	853.4	152.2
**11g**	*m-*	6-Cl	CH_3_	4521	2356	116.2	92.7
**13a**	*p-*	6-CH_3_	-	78.4	36.5	25.8	9.8
**13b**	*p-*	6-OCH_3_	-	92.1	58.4	5.5	13.2
**13c**	*p-*	6-Cl	-	55.4	7.3	18.6	8.7
**16**	-	-	-	81.4	31.1	21.7	25.4
**AAZ**	-	-	-	250	12.5	25.0	5.7

^1^ Mean from 3 different assays, using a stopped flow technique (errors were in the range of ±5–10% of the reported values).

**Table 2:** Selectivity ratios for the inhibition of hCA IX and XII over hCA I and II for target compounds **9a**–**d**, **11a**–**g**, **13a**–**c** and **16**.

cmpd	I/IX	II/IX	I/XII	II/XII
**9a**	15.3	2.2	24.5	3.4
**9b**	22.1	1.3	20.5	1.2
**9c**	18.9	1.3	32.3	2.3
**9d**	60.3	4.3	58.9	4.2
**11a**	9.5	3.8	16.1	6.4
**11b**	3.0	4.2	3.5	5.0
**11c**	52.7	18.4	8.0	2.8
**11d**	2.0	4.2	3.1	6.5
**11e**	33.8	14.5	39.7	17.1
**11f**	2.6	1.2	14.8	6.6
**11g**	38.9	20.3	48.8	25.4
**13a**	3.0	1.4	8.0	3.7
**13b**	16.7	10.6	7.0	4.4
**13c**	3.0	0.4	6.4	0.8
**16**	3.8	1.4	3.2	1.2
**AAZ**	10.0	0.5	43.9	2.2

**Table 3:** Anti-proliferative action of **QBS 11c** and **13b** against breast MDA-MB-231 and MCF-7 cancer cell lines.

Compound	IC_50_ (μM) ^1^	
	MDA-MB-231	MCF-7
**11c**	1.03 ± 0.05	0.43 ± 0.02
**13b**	2.24 ± 0.1	3.69 ± 0.17
**Doxorubicin**	1.67 ± 0.08	3.04 ± 0.14

^1^ IC_50_ values are the mean ± S.D. of three separate experiments.

**Table 4:** Effect of **QBS 11c** and **13b** on the expression levels of Bax, Bcl-2 and active Caspase-3 in breast cancer MDA-MB-231 cells.

Compound	MDA-MB-231		
	BAX (Pg/mg of Total Protein)	Bcl-2 (Pg/mg of Total Protein)	Active Caspase-3 (Pg/mg of Total Protein)
**11c**	420.9 ± 8.24 (7.1) ^1^	6.45 ± 0.17 (0.45) ^1^	12.69 ± 1.14 (4.93) ^1^
**13b**	373.5 ± 10.4 (6.3) ^1^	5.304 ± 0.13 (0.37) ^1^	9.32 ± 0.73 (3.62) ^1^
**Control**	59.43 ± 24.8	14.29 ± 0.10	2.57 ± 0.35

^1^ Numbers given between parentheses are the numbers of folds of control.

**Table 5:** Effect of **QBS 11c** and **13b** on the expression levels of Bax, Bcl-2 and active Caspase-3 in breast cancer MCF-7 cells.

Compound	MCF-7		
	BAX (Pg/mg of Total Protein)	Bcl-2 (Pg/mg of Total Protein)	Active Caspase-3 (Pg/mg of Total Protein)
**11c**	512.9 ± 11.7 (5.36) ^1^	7.29 ± 0.15 (0.79) ^1^	17.83 ± 0.62 (3.64) ^1^
**13b**	443.9 ± 10.2 (4.64) ^1^	5.72 ± 0.13 (0.62) ^1^	14.82 ± 0.43 (3.02) ^1^
**Control**	95.6 ± 11.6	9.14 ± 0.21	4.895 ± 0.66

^1^ Numbers given between parentheses are the numbers of folds of control.

**Table 6:** The docking scores and binding energy from MMGBSA for **QBS 11c** and **13b** on *h*CA IX and XII.

Comp.	*h*CA IX		*h*CA XII	
	Docking Score	MMGBSA dG Bind	Docking Score	MMGBSA dG Bind
**11c**	−6.029	−34.30	−6.029	−46.69
**13b**	−6.046	−42.14	−6.441	−49.81

## Data Availability

Not applicable.

## Supplementary Materials

The following are available online at https://www.mdpi.com/article/10.3390/ijms222011119/s1.
