# Supplementary material for: Development of Novel Quinoline-Based Sulfonamides as Selective Cancer-Associated Carbonic Anhydrase Isoform IX Inhibitors
Source: Int J Mol Sci. 2021 Oct 15;22(20):11119. doi: 10.3390/ijms222011119 (PMC8541628; doi:10.3390/ijms222011119)
Supplement: Supplementary file 1 [file ijms-22-11119-s001.zip › ESI_Biological Evaluations.pdf]

Supporting Information

Development of novel quinoline-based sulfonamides as selective cancer-associated carbonic anhydrase isoform IX inhibitors

Moataz Shaldam, Alessio Nocentini, Zainab M. Elsayed, Tamer M. Ibrahim, Rofida Salem, Ramadan A. El-Domany, Clemente Capasso, Claudiu T. Supuran*, Wagdy M. Eldehna*

Tables of Contents

1. **Carbonic Anhydrase Inhibition Assay** 2
2. **Anti-proliferative Activity** 2
3. **Assessment of Apoptotic Markers Levels** 3
4. **Molecular Modeling Study** 4
5. **IC_{50} Plots** 5-6
6. **NMR Spectra** 7-31

1. Carbonic anhydrase inhibition assay

The carbonic anhydrase catalyzed CO_2 hydration actions for all **QBS** (**9a-d**, **11a-g**, **13a-c** and **16**) herein reported have were assayed utilizing an instrument of Applied Photophysics stopped-flow. The enzymes are recombinant proteins prepared in our lab. Phenol red (at a concentration of 0.2 mM) has been used as indicator, working at the absorbance maximum of 557 nm, with 20 mM Hepes (pH 7.5) as buffer, and 20 mM Na_2SO_4 (for maintaining constant the ionic strength), following the initial rates of the CA-catalyzed CO_2 hydration reaction for a period of 10-100 s. The CO_2 concentrations ranged from 1.7 to 17 mM for the determination of the kinetic parameters and inhibition constants. For each inhibitor at least six traces of the initial 5-10% of the reaction have been used for determining the initial velocity. The uncatalyzed rates were determined in the same manner and subtracted from the total observed rates. Stock solutions of inhibitor (0.1 mM) were prepared in distilled-deionized water and dilutions up to 0.01 nM were done thereafter with the assay buffer. Inhibitor and enzyme solutions were preincubated together for 15 min at room temperature prior to assay, in order to allow for the formation of the E-I complex. The inhibition constants were obtained by non-linear least-squares methods using PRISM 3 and the Cheng-Prusoff equation, and represent the mean from at least three different determinations.

2. Anti-proliferative action toward human breast cell lines

The two examined human breast cancer cell lines (MCF-7 and MDA-MB-231) have been obtained from American Type Culture Collection (ATCC). Cells lines were maintained as monolayers in Dulbecco's Modified Eagle's Medium (DMEM) supplemented with 10% FBS, 2 mM L-glutamine, 100 U/ml penicillin and 100µg/ml streptomycin sulfate. Cobalt (II) chloride ($CoCl_2$) (100 µM) was utilized as an inducer of HIF-1α to furnish a chemically-induced hypoxia. The $CoCl_2$-induced chemical hypoxia model is one of the most commonly used hypoxia mimics. Compared to low oxygen-induced hypoxia and the use of other hypoxia mimics, the stabilization of HIF-1α and HIF-2α is sustained for several hours. So, this model allows users a wider time window to manipulate and analyze their samples under normoxic conditions. It is currently accepted that Co^{2+} substitutes Fe^{2+} in prolyl hydroxylases (PHDs), the key enzymes that link O_2 concentration to the degradation of HIF under normoxic conditions.

Cells were sub-cultured with trypsine /EDTA solution, counted with haemocytometer and plated onto 96-well plates (5000 cells/well) and left overnight to form a semi-confluent

monolayer. Cell monolayers were treated in quadrates with vehicle (DMSO, 0.1% v/v), test samples or Adriamycin as positive control for an exposure time of 72 h. At the end of exposure, MTT solution in PBS (5 mg/ml) was then added to all well including no cell blank and left to incubate for 90 min. The formation of formazan crystals were visually confirmed using phase contract microscopy. DMSO (100 μl/well) was added to dissolve the formazan crystals with shaking for 10 min after which the absorbance was read at 590 nm against no cell blanks on a FLuo Star Optima microplate reader (BMG technologies, Germany). Cell proliferation was calculated comparing the OD values of the DMSO control wells and those of the samples represented as % proliferation to the control. Dose-response experiment was performed on samples producing > or =50% loss of cell proliferation using five serial 2-fold dilutions (50, 25, 12.5, 6.25 and 3.125 μM) of the sample. IC_{50} values (concentration of sample causing 50% loss of cell proliferation of the vehicle control) were calculated using non-linear regression curve fitting of the dose response plots on GraphPad Prism V.6.0 software.

3. Assessment of Apoptotic Markers Levels

The levels of the pro-apoptotic markers (Bax and active caspase-3) as well as the anti-apoptotic marker Bcl-2 were determined using ELISA kits; Human Bax ELISA Kit (EIA-4487) DRG® Instruments, Bcl-2 ELISA Kit (99–0042), Zymed® Laboratories, Invitrogen Immunodetection, and Human Caspase-3 (active) ELISA Kit (KHO1091) Invitrogen®, per the manufacturer's instructions. Breast cancer MDA-MB-231 and MCF-7 cells were cultured as a monolayer in T-25 flasks and were seeded to attain 30% confluency prior to treatment. Cells were then treated separately with MBFS **11b** at its IC_{50} concentration for 24 h. At the end of treatment, cells were collected via trypsinization and centrifuged at 10,000 rpm. The pellet was then rinsed with PBS and lysed in RIPA lysis buffer at 4 °C for 45 min, then centrifuged at 14,000 rpm for 20 min to remove the cellular debris. Lysates were then collected and stored at −80 °C for later protein determination using Pierce BCA Protein Assay Kit according to manufacturer's recommendations.

The cell lysate was diluted 10 times, and 100 μL (50 mg protein) was added to the wells of four separate microtiter plates for the three ELISA kits that were pre-coated with primary antibodies specific to Bax, Bcl-2, and caspase-3 proteins, respectively. A secondary biotin-linked antibody specific to the protein captured by the primary antibody was further added to bind the

captured protein, forming a "sandwich" of specific antibodies around the desired protein in the cell lysate. The streptavidin-HRP complex was then used to bind the biotin-linked secondary antibody through its streptavidin portion. The HRP domain reacted with the added TMB substrate to form a colored product that measured at 450 nm by a plate reader ChroMate-4300 after the reaction was terminated *via* the addition of stop solution.

4. Molecular Modeling Study

The crystal structure of hCA IX (PDB 5FL4) and hCA XII (PDB 4WW8) were retrieved from protein data bank and water molecules were ignored. The Protein Preparation Wizard tool in Maestro was used to convey bond orders, add hydrogens, optimize H-bonds and minimize energy using Optimized Potentials for Liquid Simulation (OPLS3) force field with a root mean square deviation (RMSD) value of 0.30. The ligand 3D structures were prepared by Maestro and their ionization states was checked with Epik at pH 7.4±0.5 then they were subjected to energy minimization using OPLS3 force field in Macromodel with a maximum number of 2500 conjugate gradient iteration and a convergence of 0.05 kcal mol^{-1} A^{-1}. Glide with default parameters was used to generate the docking grid setting the center of the co-crystallized ligands as grid center. Docking was performed using the standard precision mode (SP) implemented in Glide and the top 5 poses for each ligand were returned. Prime MMGBSA was used for the refinement of the best pose for each ligand, using VSGB solvation model setting the receptor flexible within 3 A around the ligand.

5. IC$_{50}$ Plots

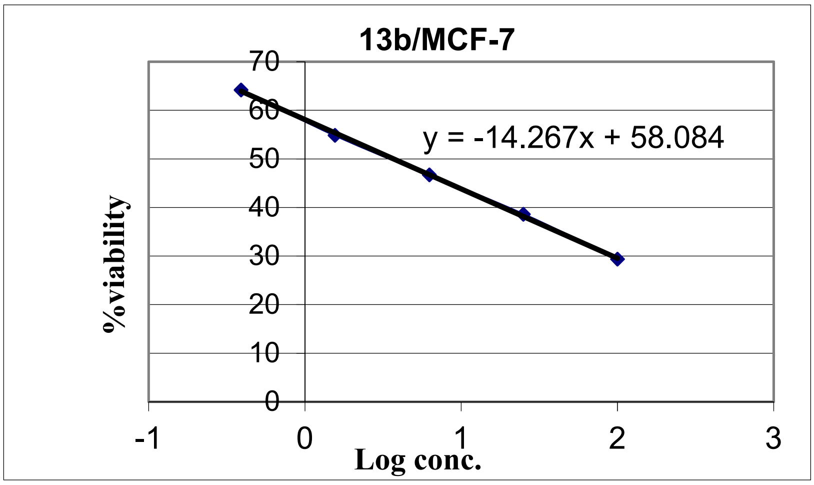

Effect of **QBS 13b** on MCF-7 cancer cell

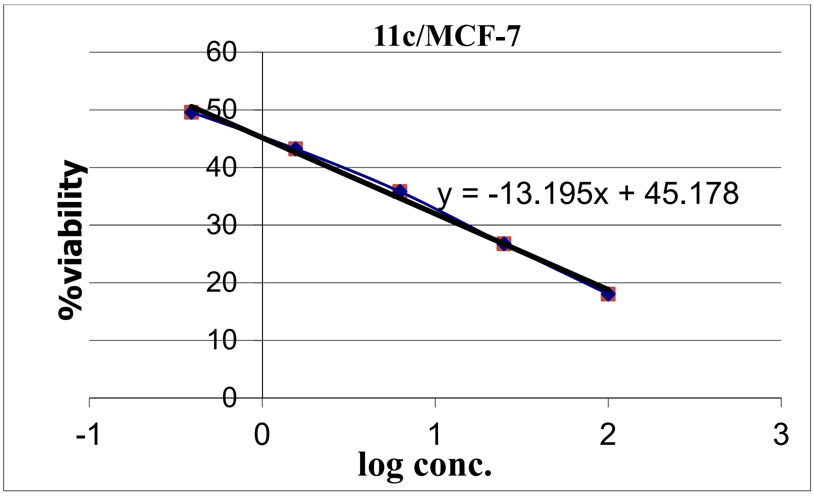

Effect of **QBS 11c** on MCF-7 cancer cell

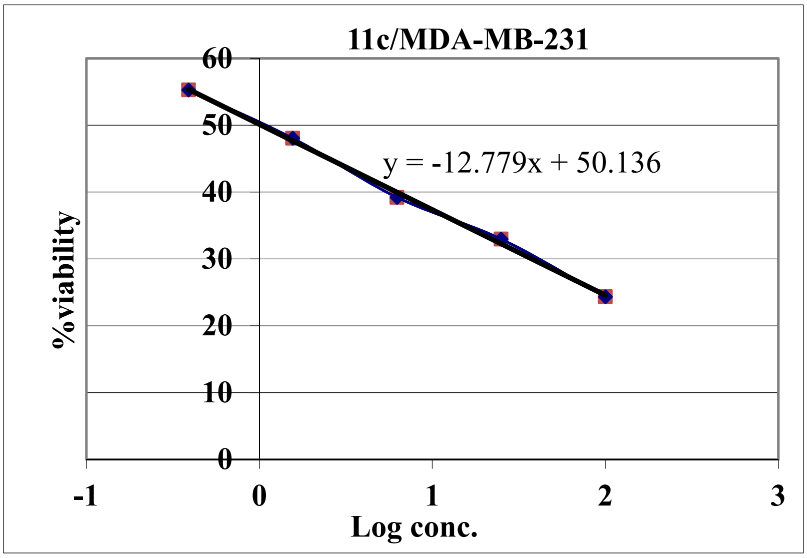

Effect of **QBS 11c** on MDA-MB-231 cancer cell

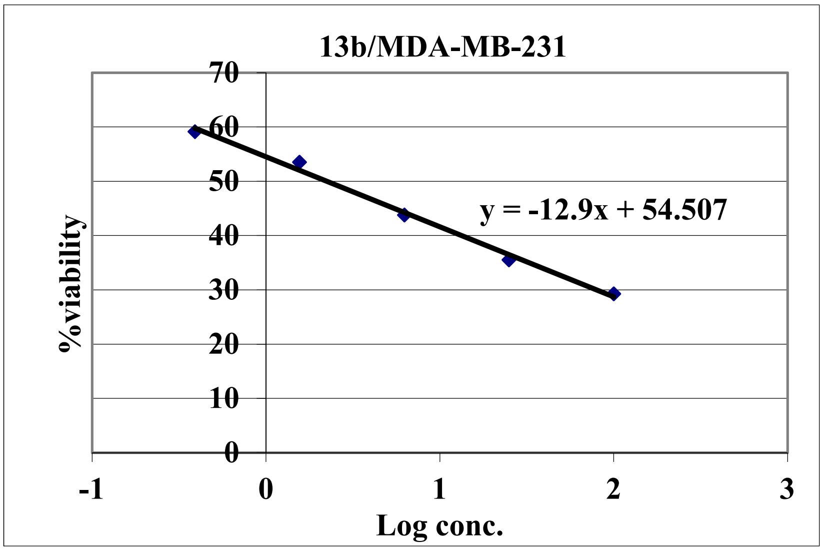

Effect of **QBS 13b** on MDA-MB-231 cancer cell
