# Supplementary material for: Development of Novel Quinoline-Based Sulfonamides as Selective Cancer-Associated Carbonic Anhydrase Isoform IX Inhibitors
Source: Int J Mol Sci. 2021 Oct 15;22(20):11119. doi: 10.3390/ijms222011119 (PMC8541628; doi:10.3390/ijms222011119)
Supplement: Supplementary file 1 [file ijms-22-11119-s001.zip › NMR charts.pdf]

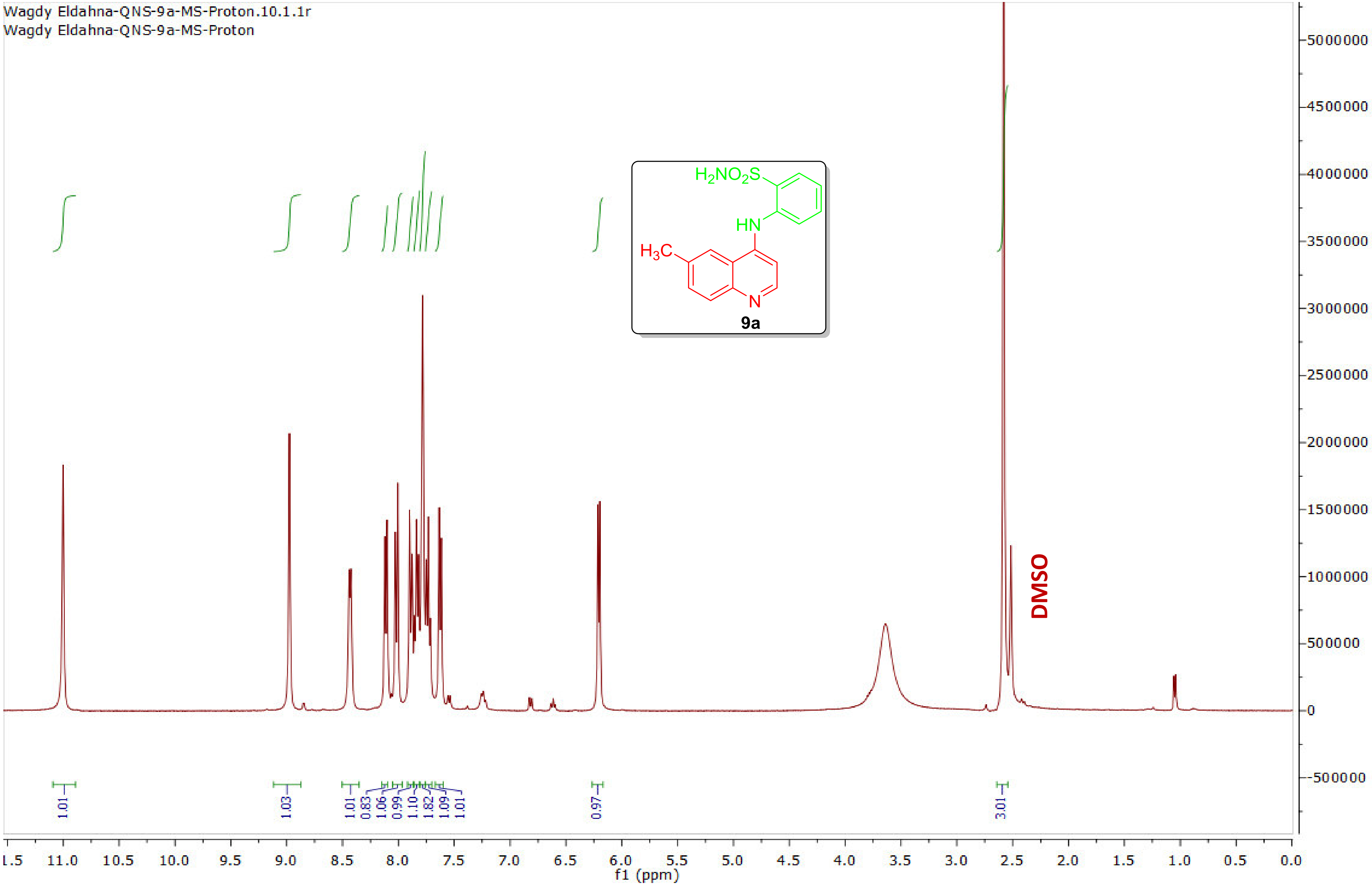
Wagdy Eldahna-QNS-9a-MS-Proton.10.1.1r
Wagdy Eldahna-QNS-9a-MS-Proton
H2NO2S
HN
H3C
N
9a
DMSO
1.01
1.03
1.01
0.83
1.06
0.99
1.10
1.82
1.09
1.01
0.97
3.01
f1 (ppm)

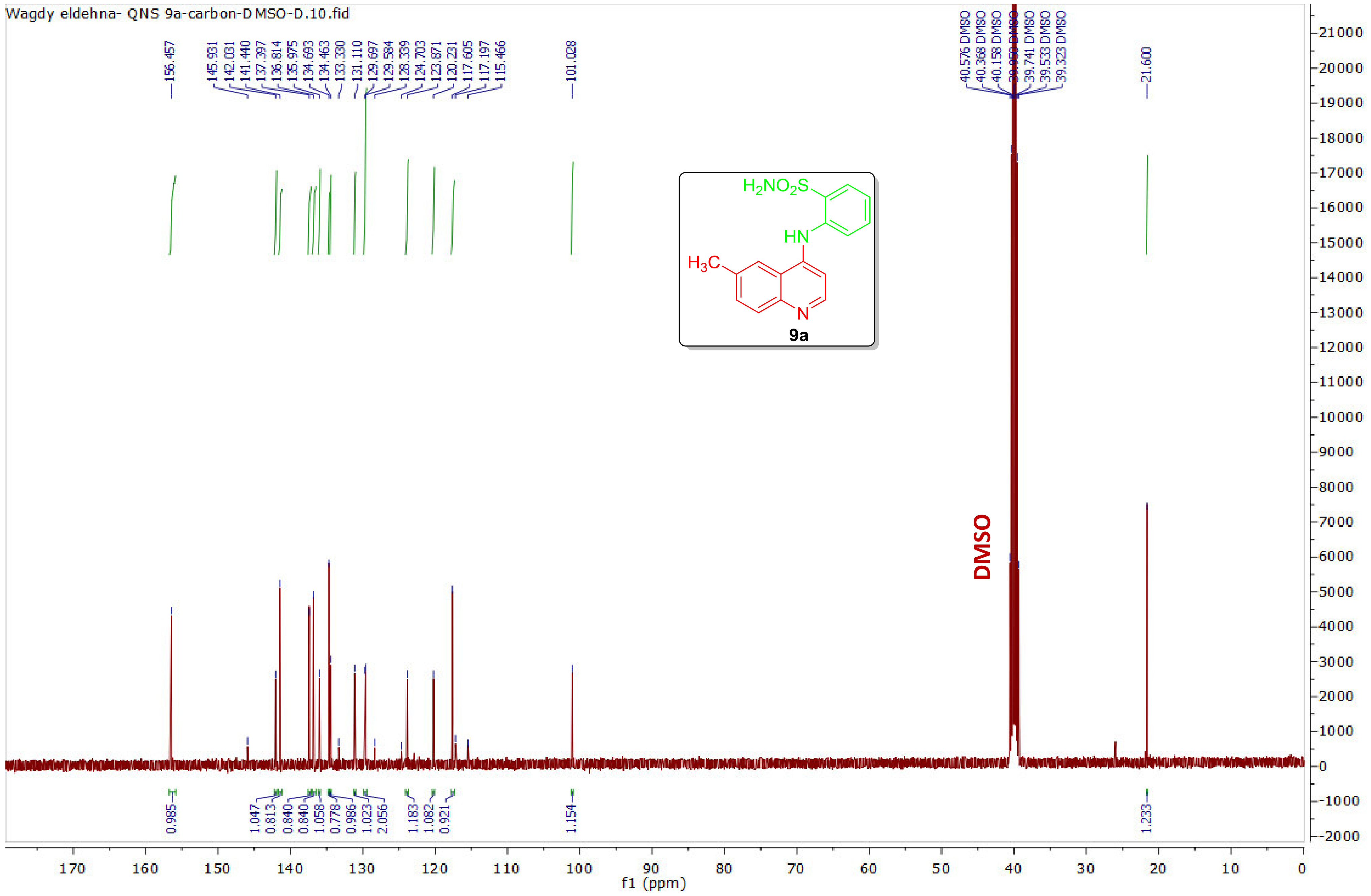
Wagdy eldehna- QNS 9a-carbon-DMSO-D.10.fid
156.457
145.931
142.031
141.440
137.397
136.814
135.975
134.693
134.463
133.330
131.110
129.697
129.584
128.339
124.703
123.871
120.231
117.605
117.197
115.466
101.028
40.576 DMSO
40.368 DMSO
40.158 DMSO
39.950 DMSO
39.741 DMSO
39.533 DMSO
39.323 DMSO
21.600
DMSO
f1 (ppm)
9a

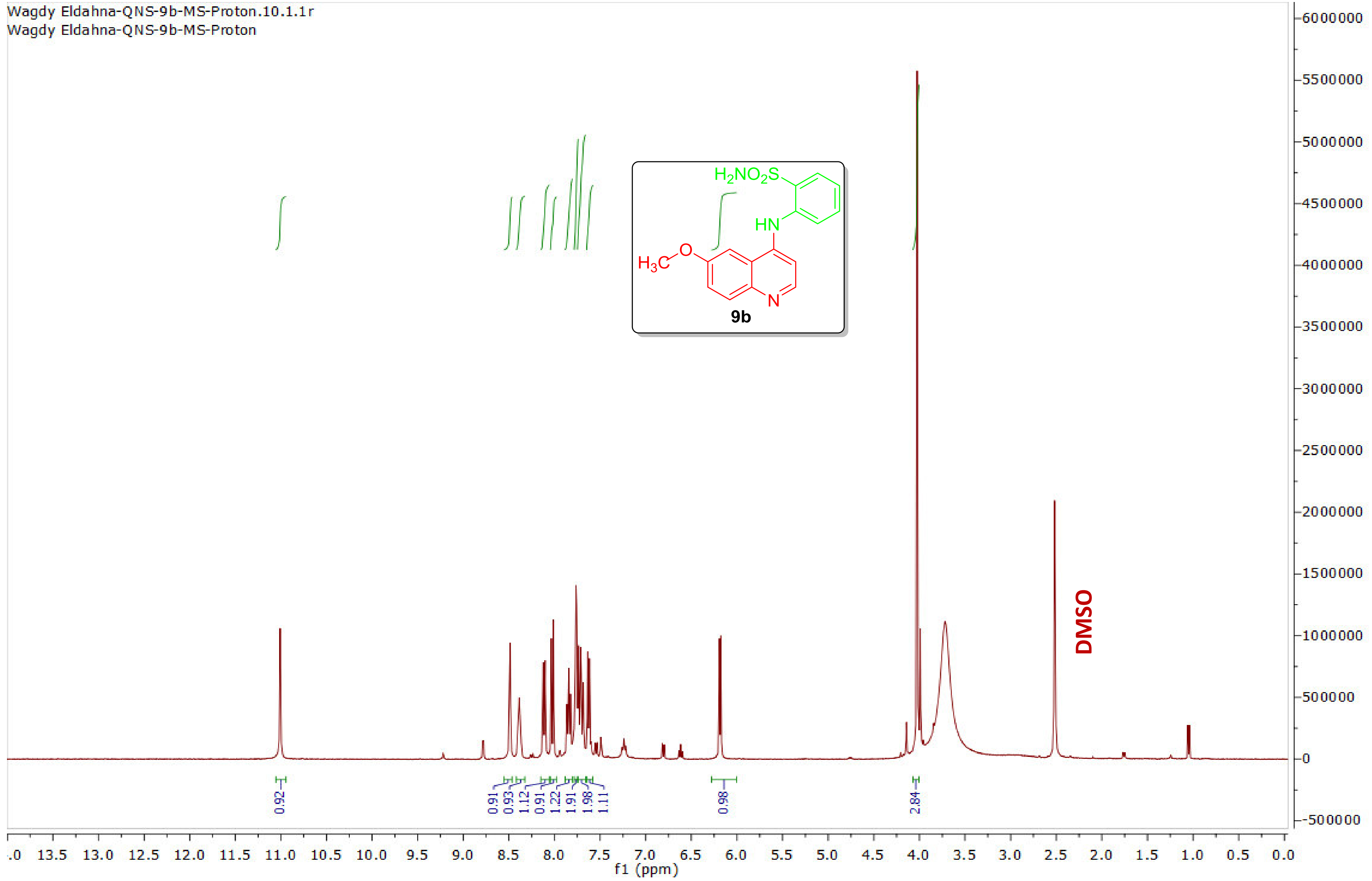
Wagdy Eldahna-QNS-9b-MS-Proton.10.1.1r
Wagdy Eldahna-QNS-9b-MS-Proton
H_2NO_2S
HN
H_3C
O
N
9b
DMSO
0.92
0.91
0.93
1.12
0.91
1.22
1.91
1.98
1.11
0.98
2.84
f1 (ppm)

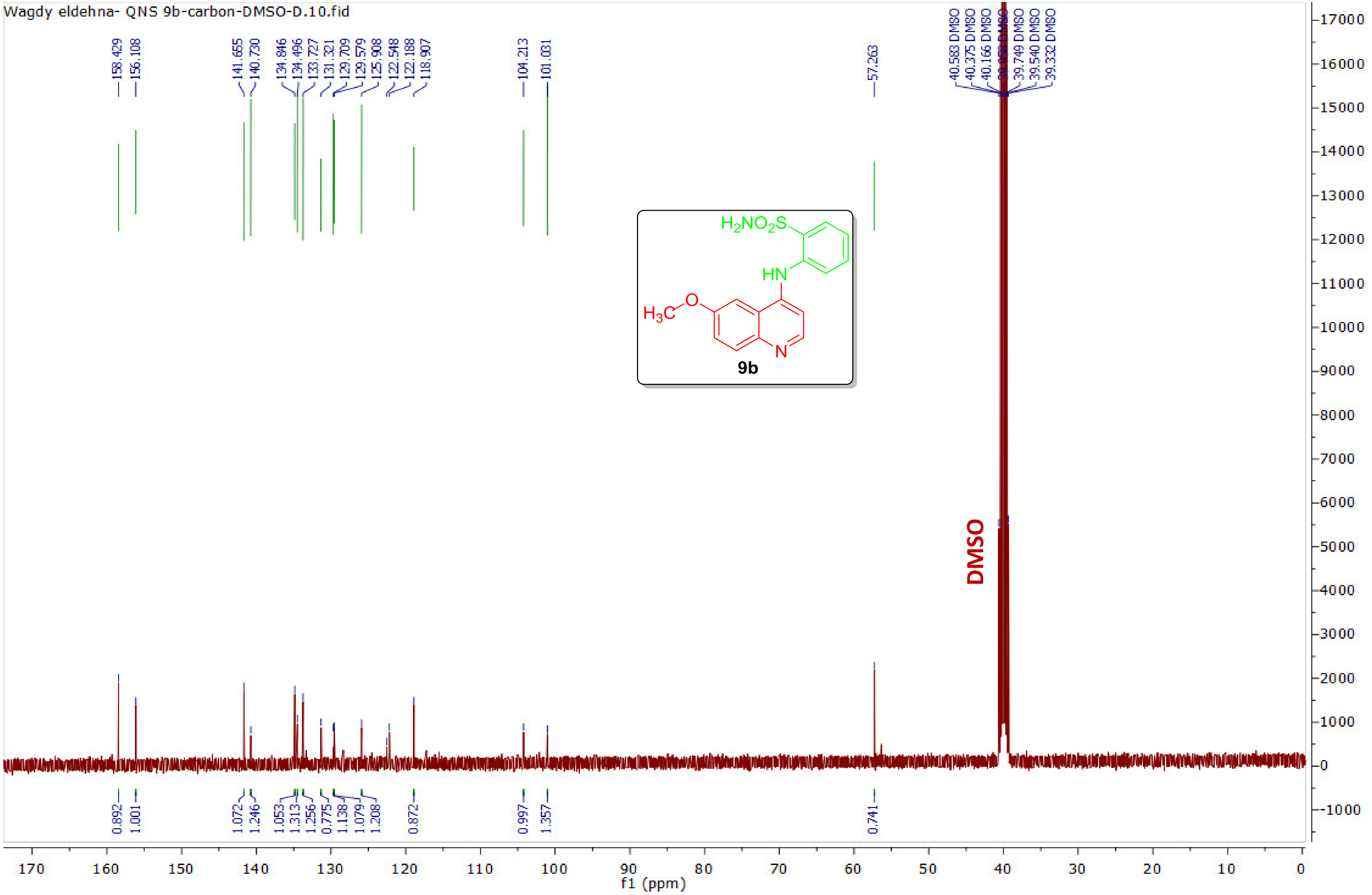
Wagdy eldehna- QNS 9b-carbon-DMSO-D.10.fid
158.429
156.108
141.655
140.730
134.846
134.496
133.727
131.321
129.709
129.579
125.908
122.548
122.188
118.907
104.213
101.031
57.263
40.583 DMSO
40.375 DMSO
40.166 DMSO
39.749 DMSO
39.540 DMSO
39.332 DMSO
H_2NO_2S
HN
H_3C
O
N
9b
DMSO
0.892
1.001
1.072
1.246
1.053
1.313
1.256
0.775
1.138
1.079
1.208
0.872
0.997
1.357
0.741
f1 (ppm)

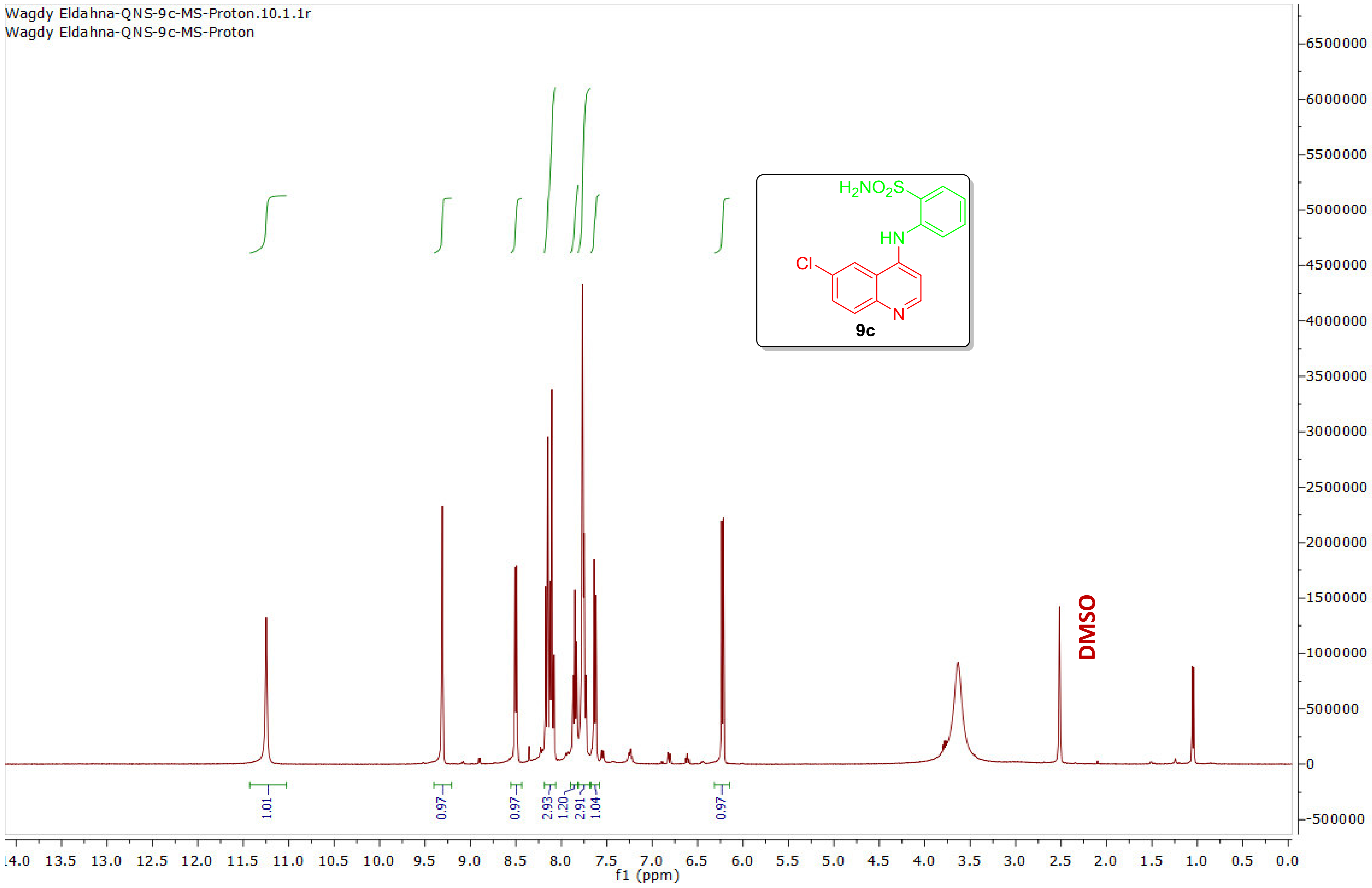

Wagdy Eldahna-QNS-9c-MS-Proton.10.1.1r
Wagdy Eldahna-QNS-9c-MS-Proton
H_2NO_2S
HN
Cl
N
9c
DMSO
1.01
0.97
0.97
2.93
1.20
2.91
1.04
0.97
f1 (ppm)

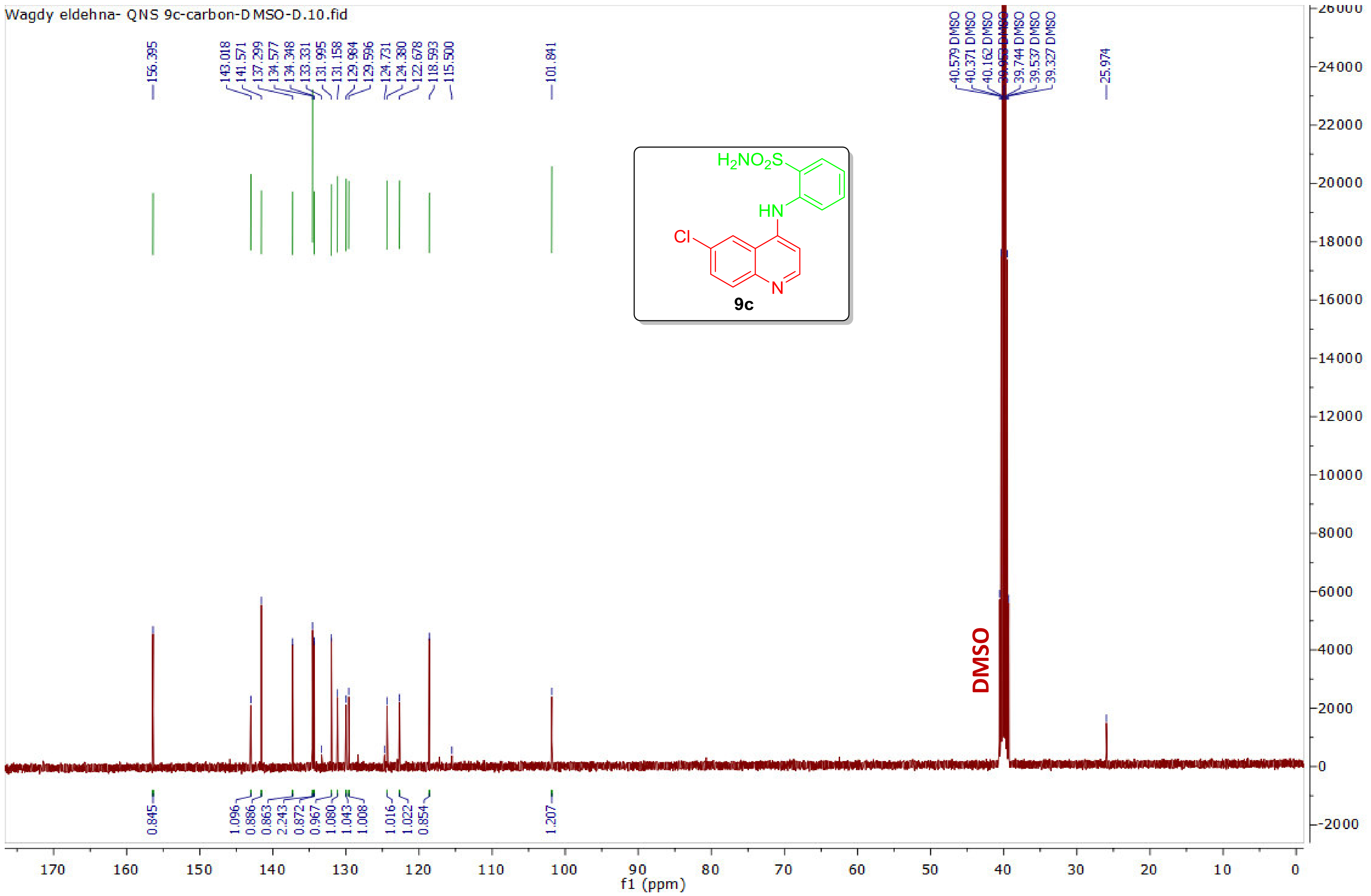
Wagdy eldehna- QNS 9c-carbon-DMSO-D.10.fid
156.395
143.018
141.571
137.299
134.577
134.348
133.331
131.995
131.158
129.984
129.596
124.731
124.380
122.678
118.593
115.500
101.841
40.579 DMSO
40.371 DMSO
40.162 DMSO
39.744 DMSO
39.537 DMSO
39.327 DMSO
25.974
H_2NO_2S
HN
Cl
N
9c
DMSO
0.845
1.096
0.886
0.863
2.243
0.872
0.967
1.080
1.043
1.008
1.016
1.022
0.854
1.207
f1 (ppm)

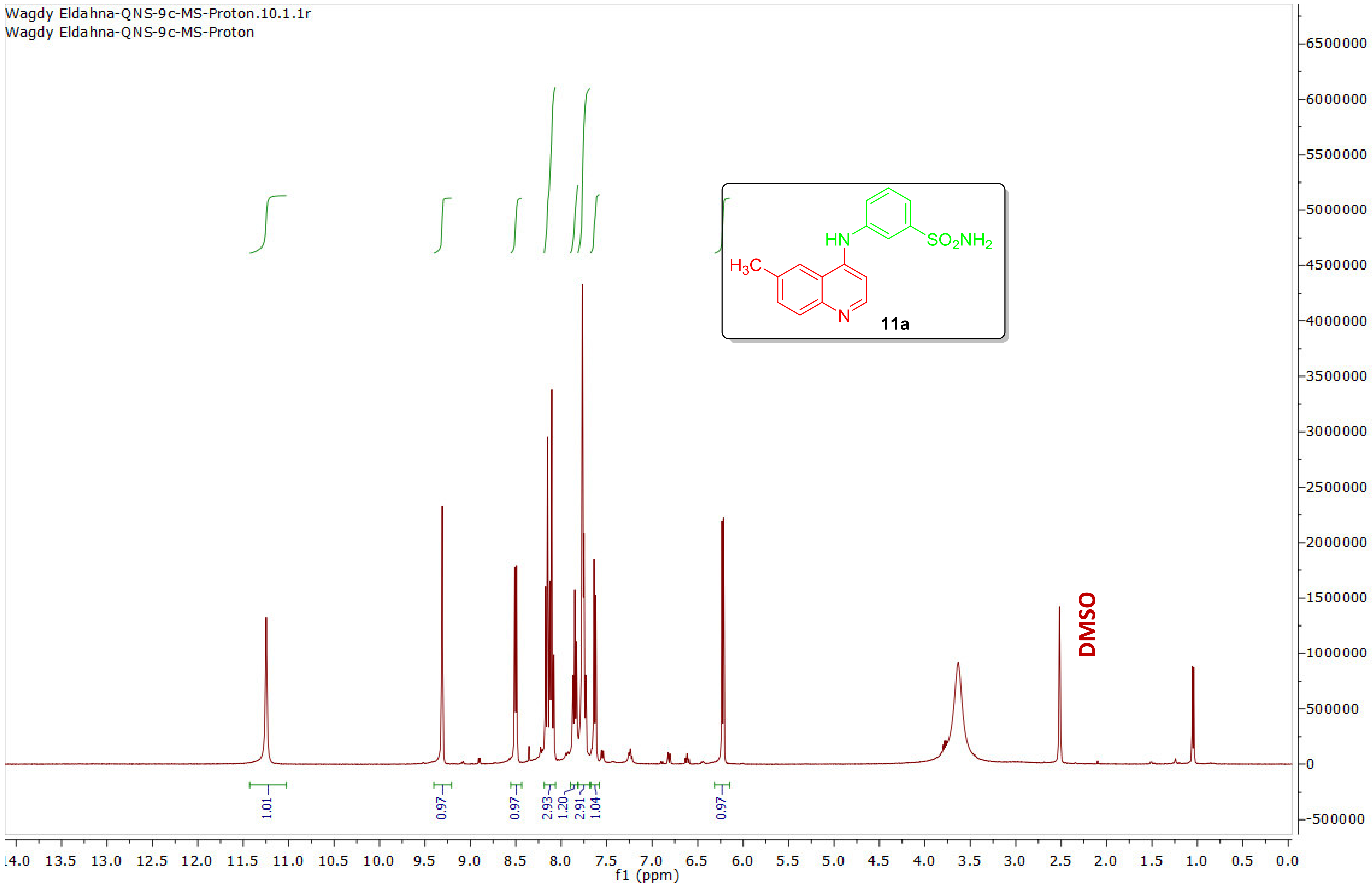

Wagdy Eldahna-QNS-9c-MS-Proton.10.1.1r
Wagdy Eldahna-QNS-9c-MS-Proton
HN
SO2NH2
H3C
N
11a
DMSO
1.01
0.97
0.97
2.93
1.20
2.91
1.04
0.97
f1 (ppm)

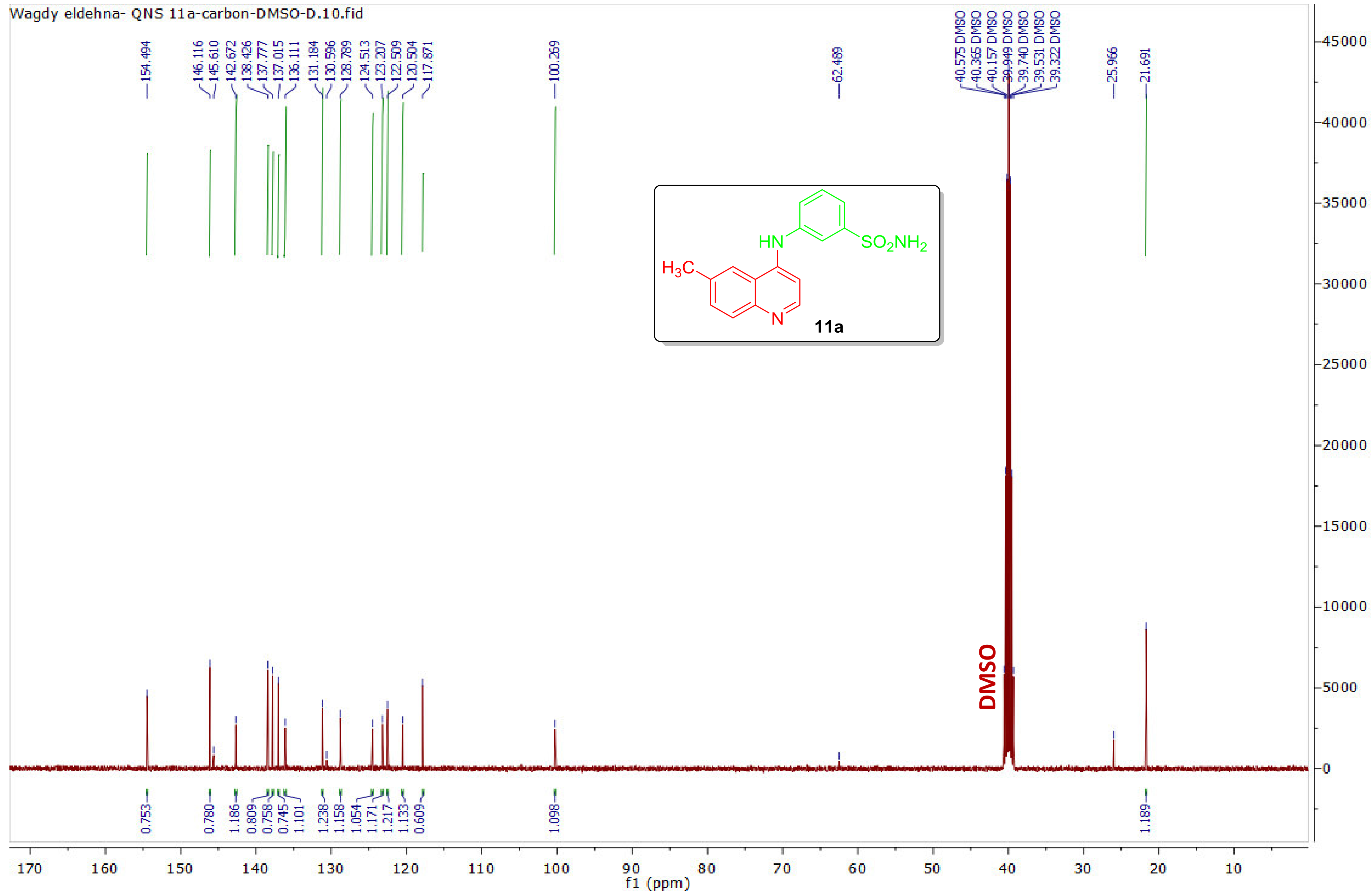

Wagdy eldehna- QNS 11a-carbon-DMSO-D.10.fid
154.494
146.116
145.610
142.672
138.426
137.777
137.015
136.111
131.184
130.596
128.789
124.513
123.207
122.509
120.504
117.871
100.269
62.489
40.575 DMSO
40.365 DMSO
40.157 DMSO
39.949 DMSO
39.740 DMSO
39.531 DMSO
39.322 DMSO
25.966
21.691
H3C
HN
SO2NH2
N
11a
DMSO
0.753
0.780
1.186
0.809
0.758
0.745
1.101
1.238
1.158
1.054
1.171
1.217
1.133
0.609
1.098
1.189
f1 (ppm)

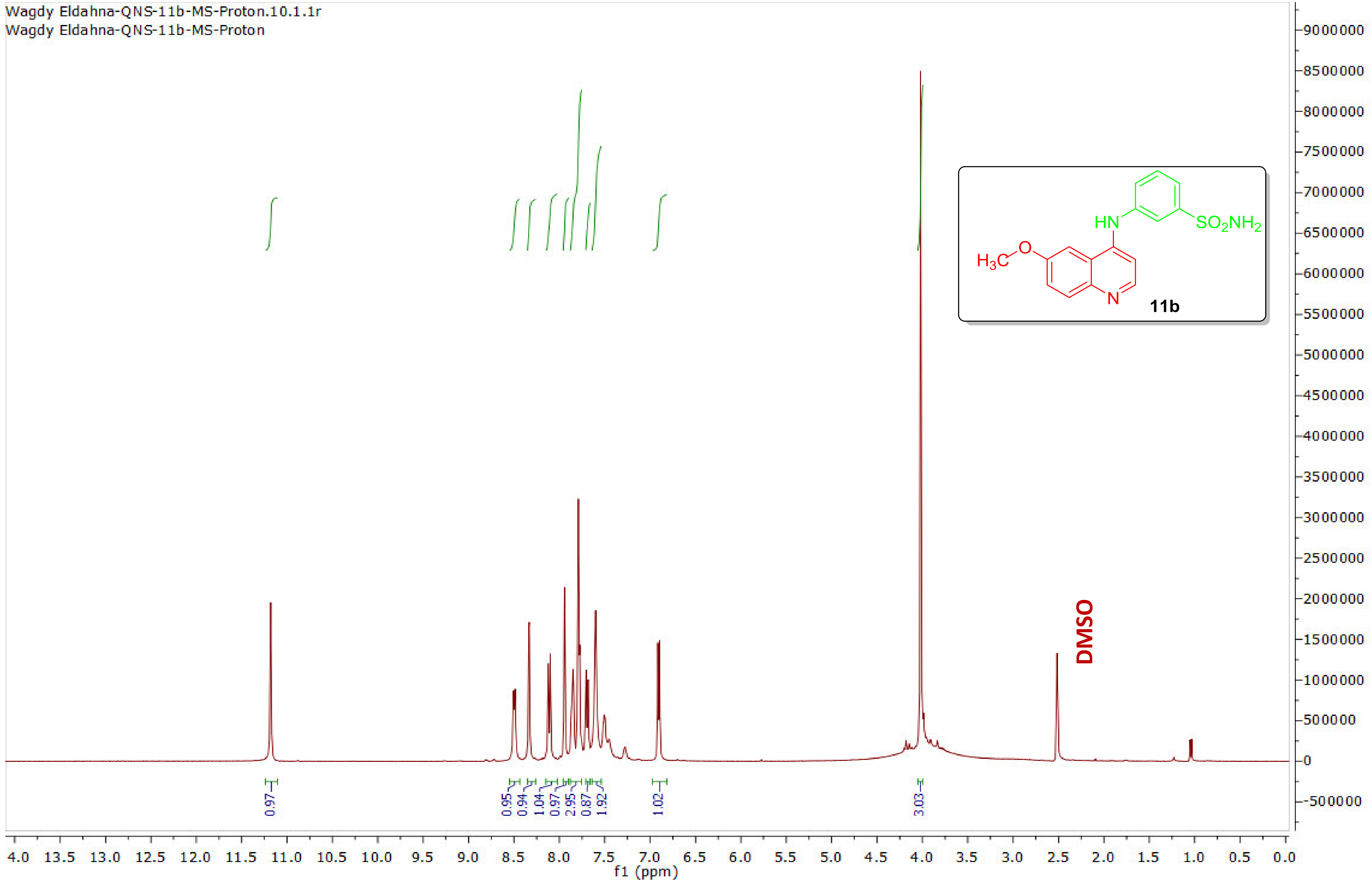
Wagdy Eldahna-QNS-11b-MS-Proton.10.1.1r
Wagdy Eldahna-QNS-11b-MS-Proton
HN
SO2NH2
H3C
O
N
11b
DMSO
0.97
0.95
0.94
1.04
0.97
2.95
0.87
1.92
1.02
3.03
f1 (ppm)

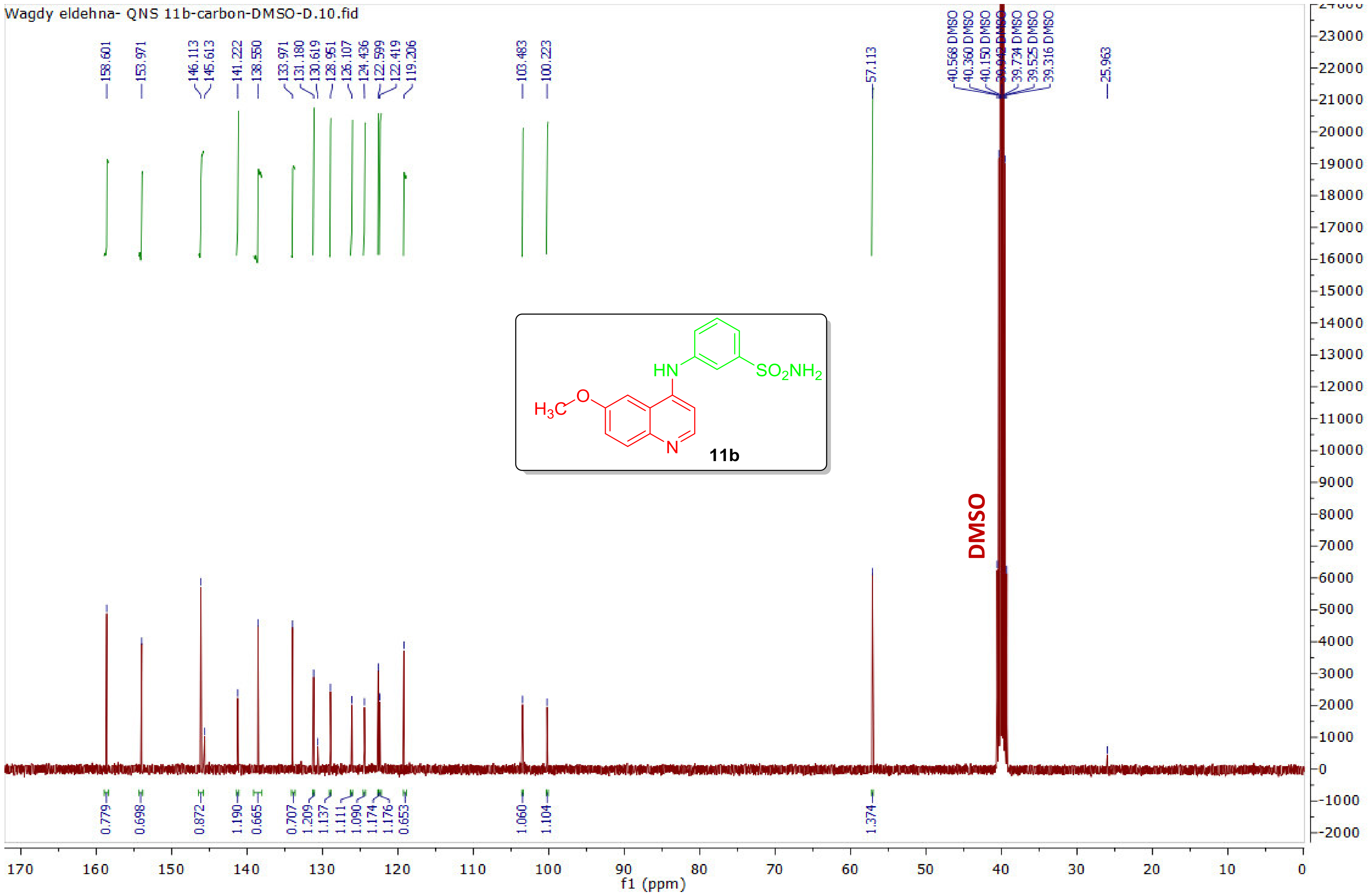

Wagdy eldehna- QNS 11b-carbon-DMSO-D.10.fid
158.601
153.971
146.113
145.613
141.222
138.550
133.971
131.180
130.619
128.951
126.107
124.436
122.599
122.419
119.206
103.483
100.223
57.113
40.568 DMSO
40.360 DMSO
40.150 DMSO
39.734 DMSO
39.525 DMSO
39.316 DMSO
25.963
HN
SO_2NH_2
H_3C
O
N
11b
DMSO
f1 (ppm)

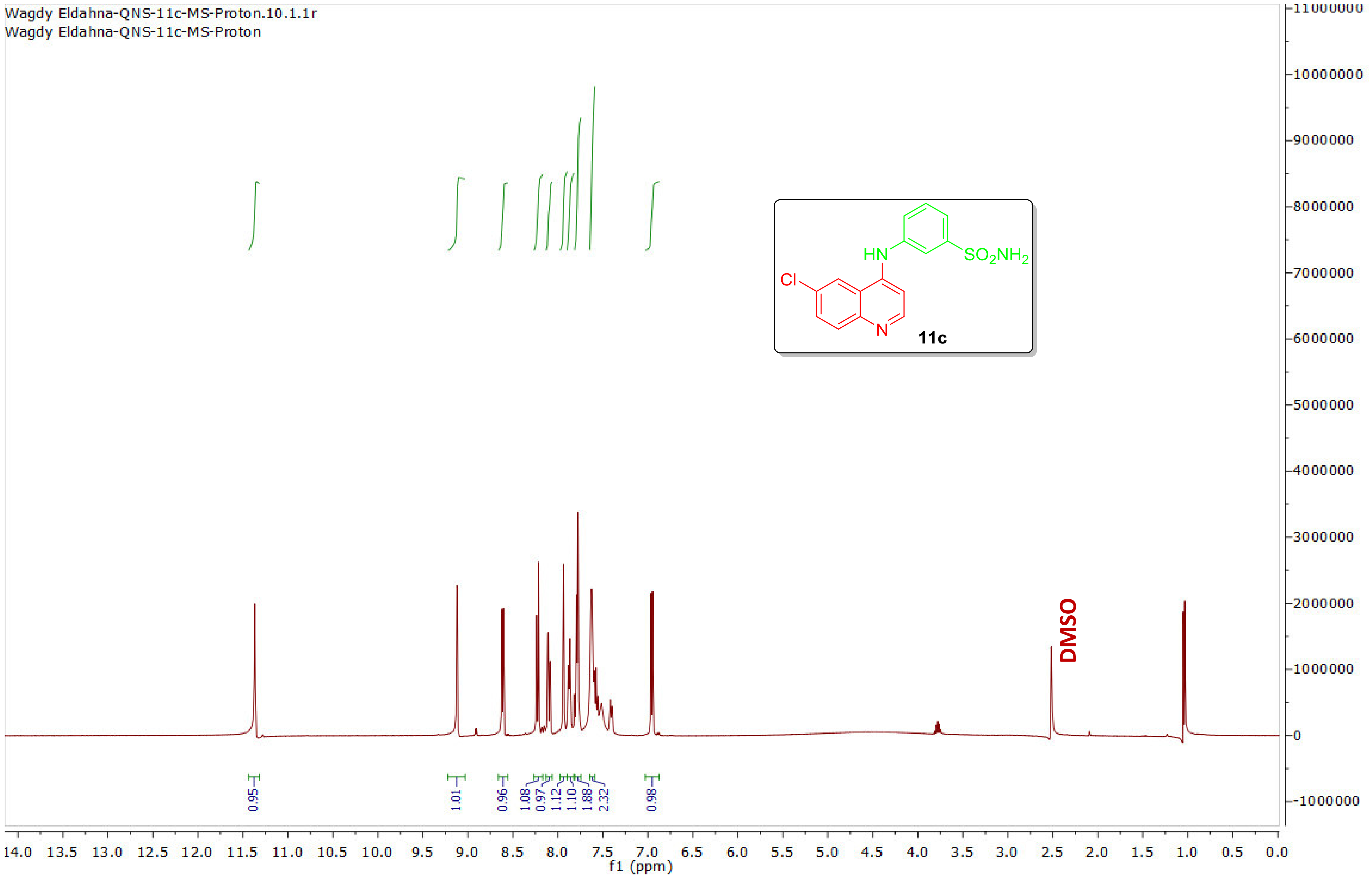

Wagdy Eldahna-QNS-11c-MS-Proton.10.1.1r
Wagdy Eldahna-QNS-11c-MS-Proton
HN
SO2NH2
Cl
N
11c
DMSO
0.95
1.01
0.96
1.08
0.97
1.12
1.10
1.88
2.32
0.98
f1 (ppm)

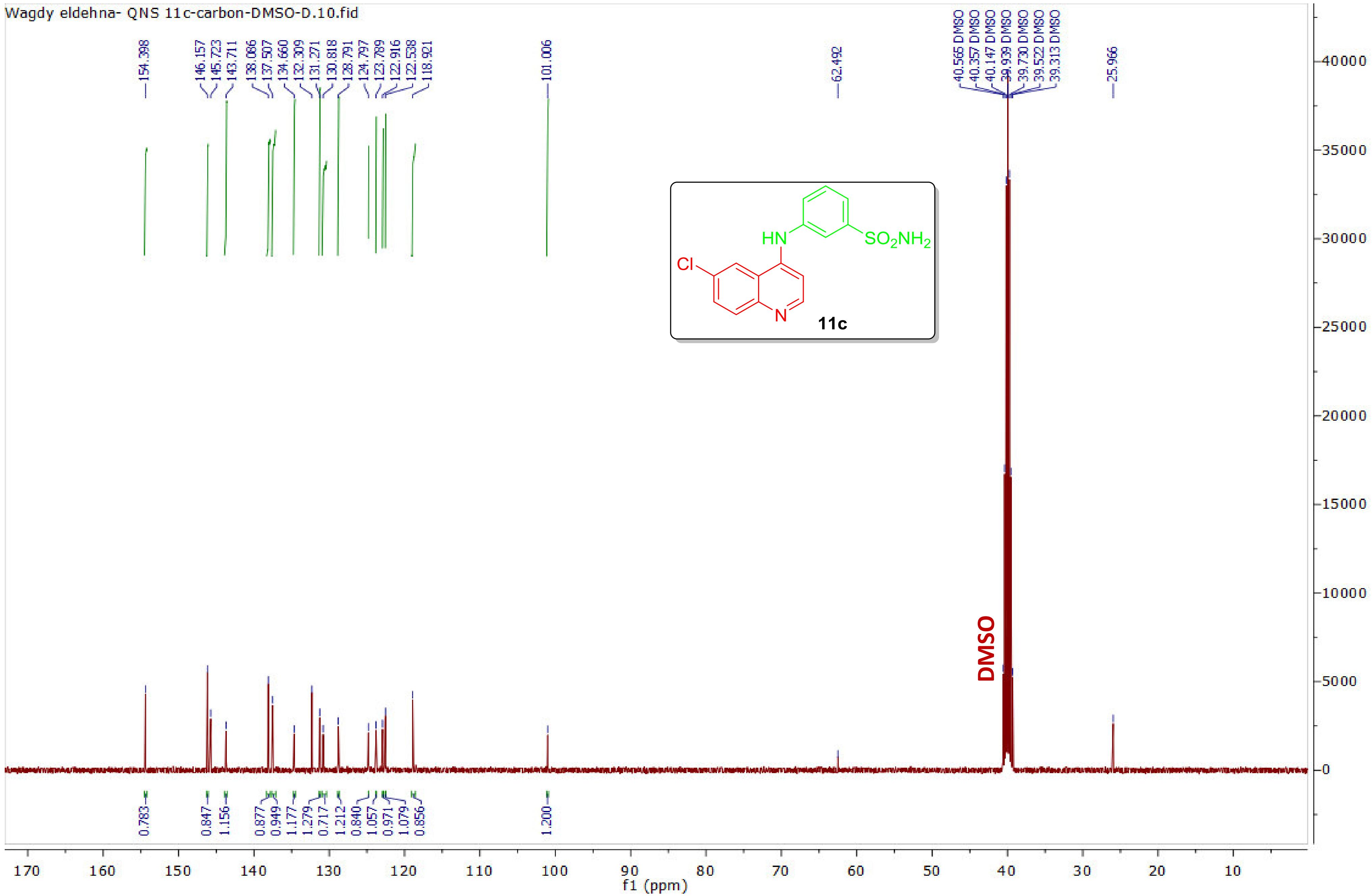
Wagdy eldehna- QNS 11c-carbon-DMSO-D.10.fid
154.398
146.157
145.723
143.711
138.086
137.507
134.660
132.309
131.271
130.818
128.791
124.797
123.789
122.916
122.538
118.921
101.006
62.492
40.565 DMSO
40.357 DMSO
40.147 DMSO
39.939 DMSO
39.730 DMSO
39.522 DMSO
39.313 DMSO
25.966
0.783
0.847
1.156
0.877
0.949
1.177
1.279
0.717
1.212
0.840
1.057
0.971
1.079
0.856
1.200
Cl
HN
SO2NH2
N
11c
DMSO
f1 (ppm)

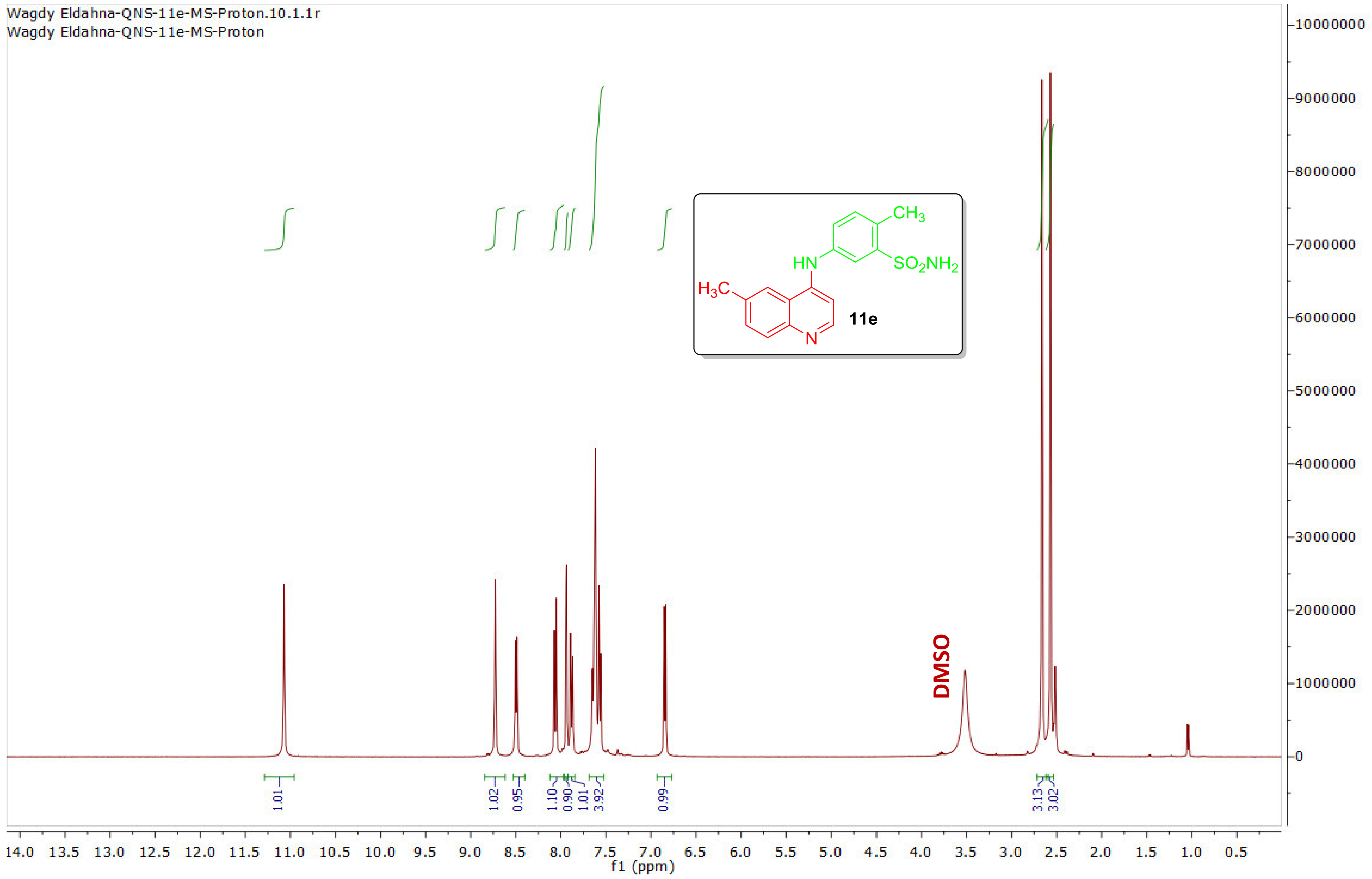
Wagdy Eldahna-QNS-11e-MS-Proton.10.1.1r
Wagdy Eldahna-QNS-11e-MS-Proton
CH3
HN
SO2NH2
H3C
N
11e
DMSO
1.01
1.02
0.95
1.10
0.90
1.01
3.92
0.99
3.13
3.02
f1 (ppm)

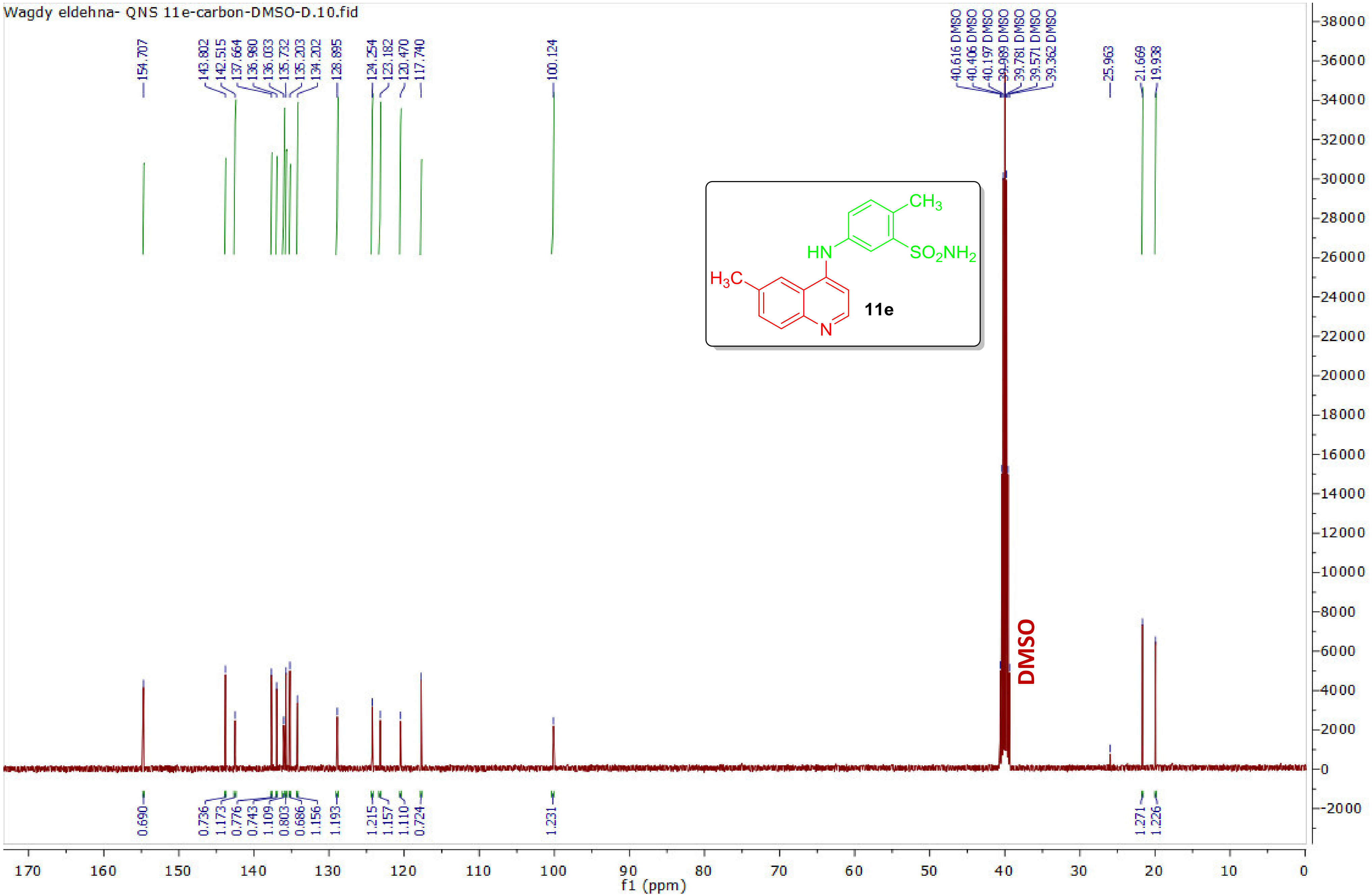

Wagdy eldehna- QNS 11e-carbon-DMSO-D.10.fid
154.707
143.802
142.515
137.664
136.980
136.033
135.732
135.203
134.202
128.895
124.254
123.182
120.470
117.740
100.124
40.616 DMSO
40.406 DMSO
40.197 DMSO
39.989 DMSO
39.781 DMSO
39.571 DMSO
39.362 DMSO
25.963
21.669
19.938
CH3
HN
SO2NH2
H3C
N
11e
DMSO
f1 (ppm)

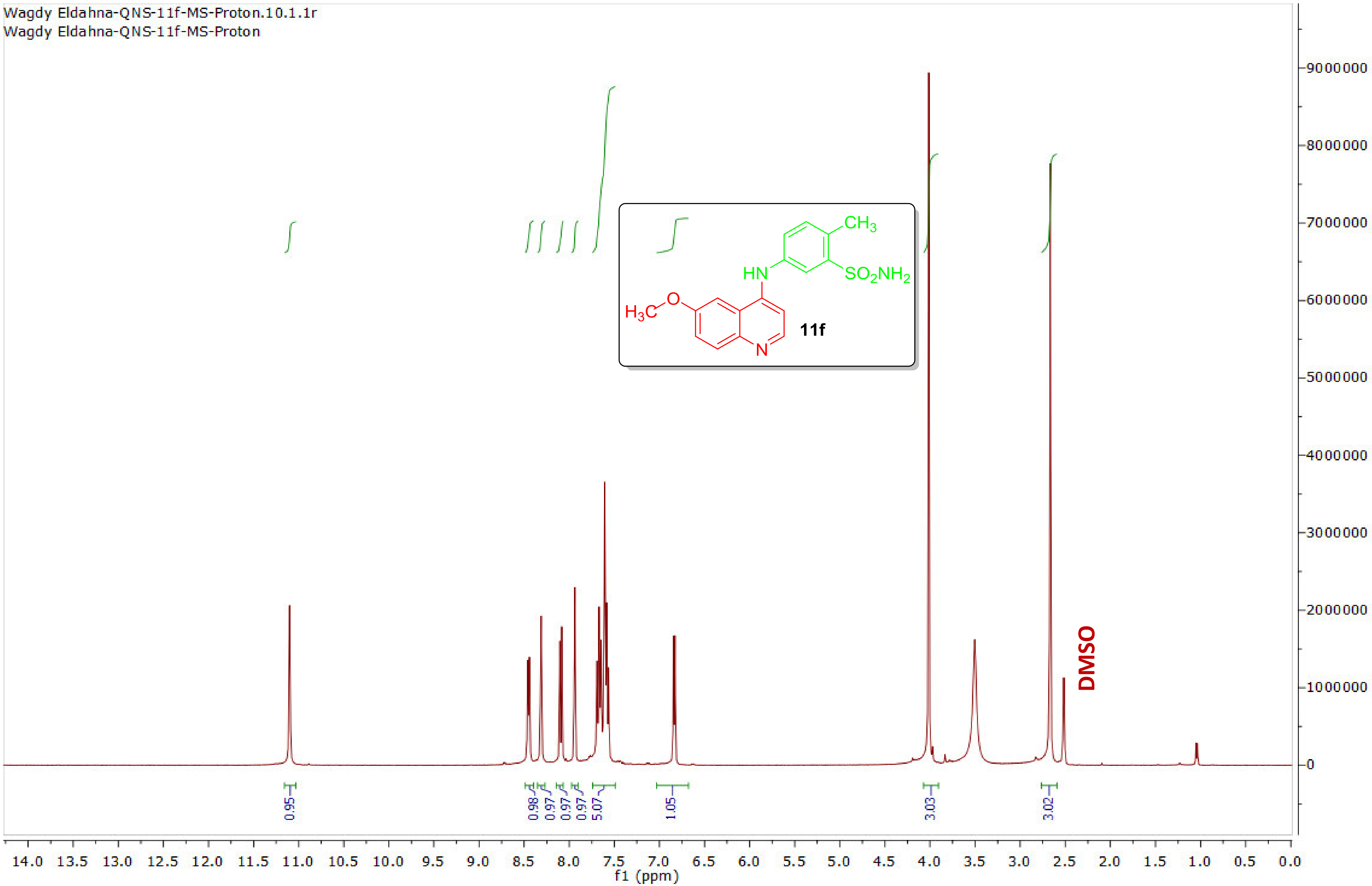

Wagdy Eldahna-QNS-11f-MS-Proton.10.1.1r
Wagdy Eldahna-QNS-11f-MS-Proton
11f
DMSO
0.95
0.98
0.97
0.97
0.97
5.07
1.05
3.03
3.02
14.0 13.5 13.0 12.5 12.0 11.5 11.0 10.5 10.0 9.5 9.0 8.5 8.0 7.5 7.0 6.5 6.0 5.5 5.0 4.5 4.0 3.5 3.0 2.5 2.0 1.5 1.0 0.5 0.0
f1 (ppm)
9000000
8000000
7000000
6000000
5000000
4000000
3000000
2000000
1000000
0

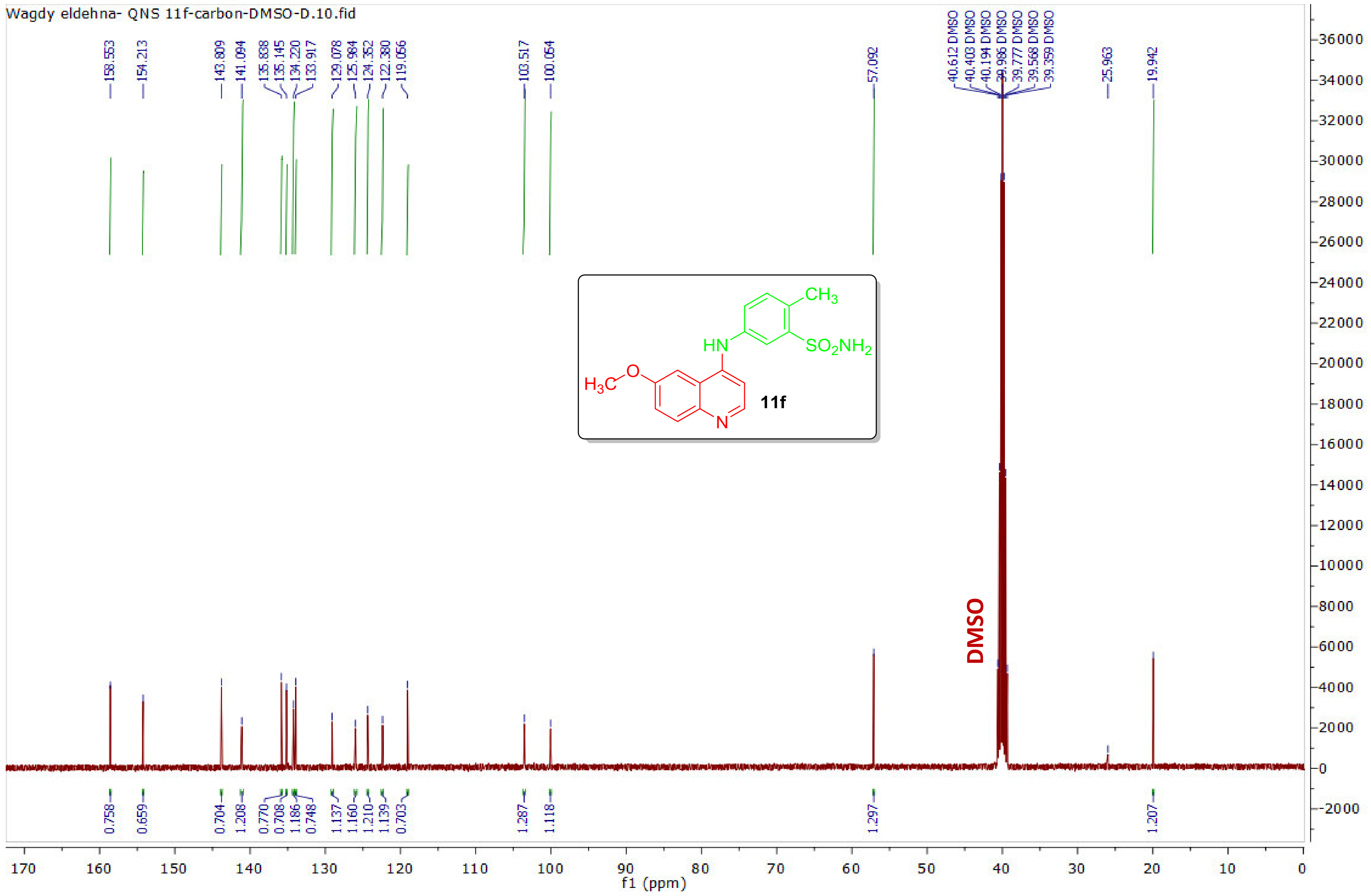

Wagdy eldehna- QNS 11f-carbon-DMSO-D.10.fid
158.553
154.213
143.809
141.094
135.838
135.145
134.220
133.917
129.078
125.984
124.352
122.380
119.056
103.517
100.054
57.092
40.612 DMSO
40.403 DMSO
40.194 DMSO
39.986 DMSO
39.777 DMSO
39.568 DMSO
39.359 DMSO
25.963
19.942
DMSO
11f
HN
CH3
SO2NH2
H3C
O
N
f1 (ppm)

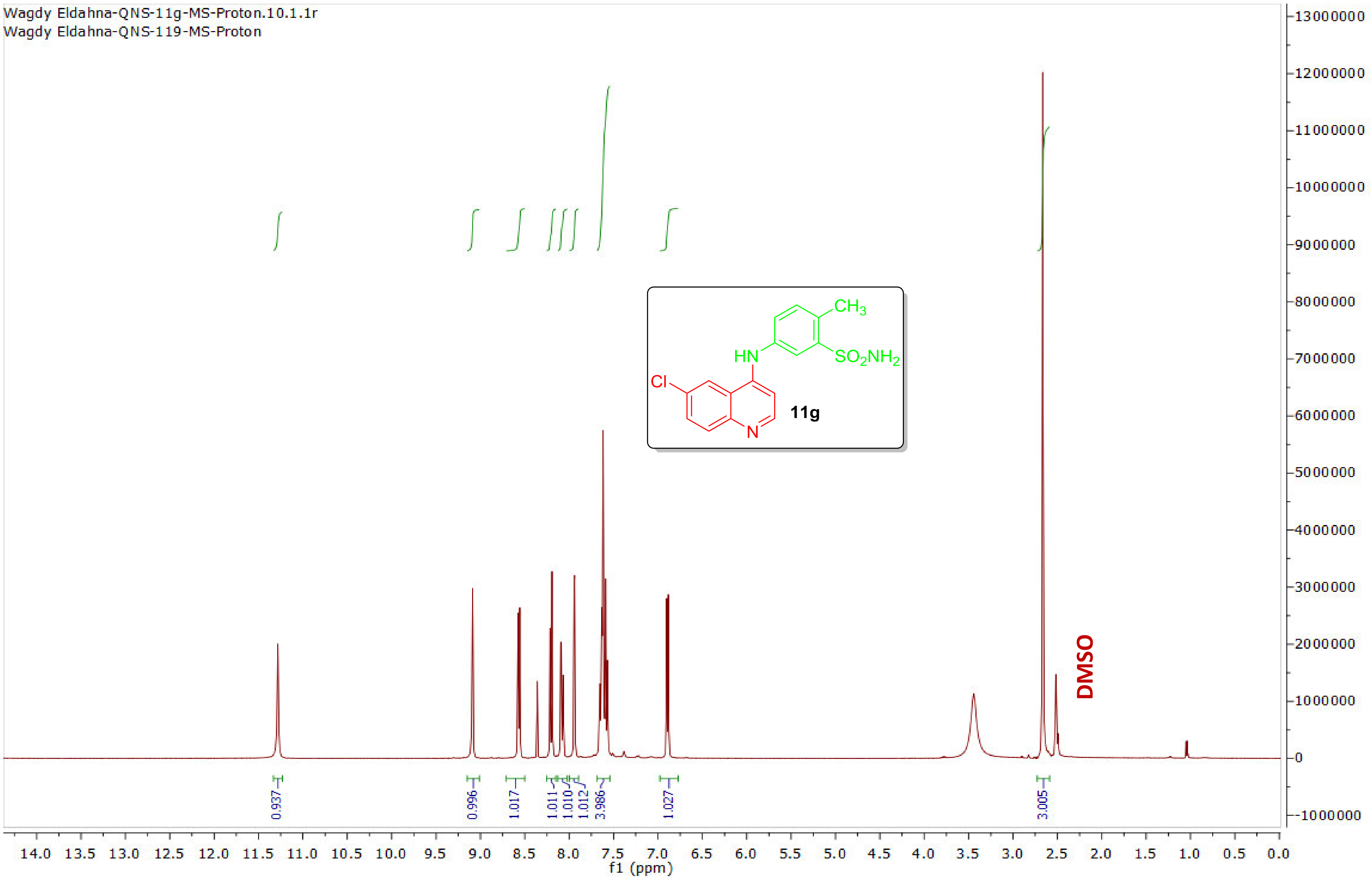
Wagdy Eldahna-QNS-11g-MS-Proton.10.1.1r
Wagdy Eldahna-QNS-119-MS-Proton
CH3
HN
SO2NH2
Cl
N
11g
DMSO
0.937
0.996
1.017
1.011
1.010
1.012
3.986
1.027
3.005
f1 (ppm)

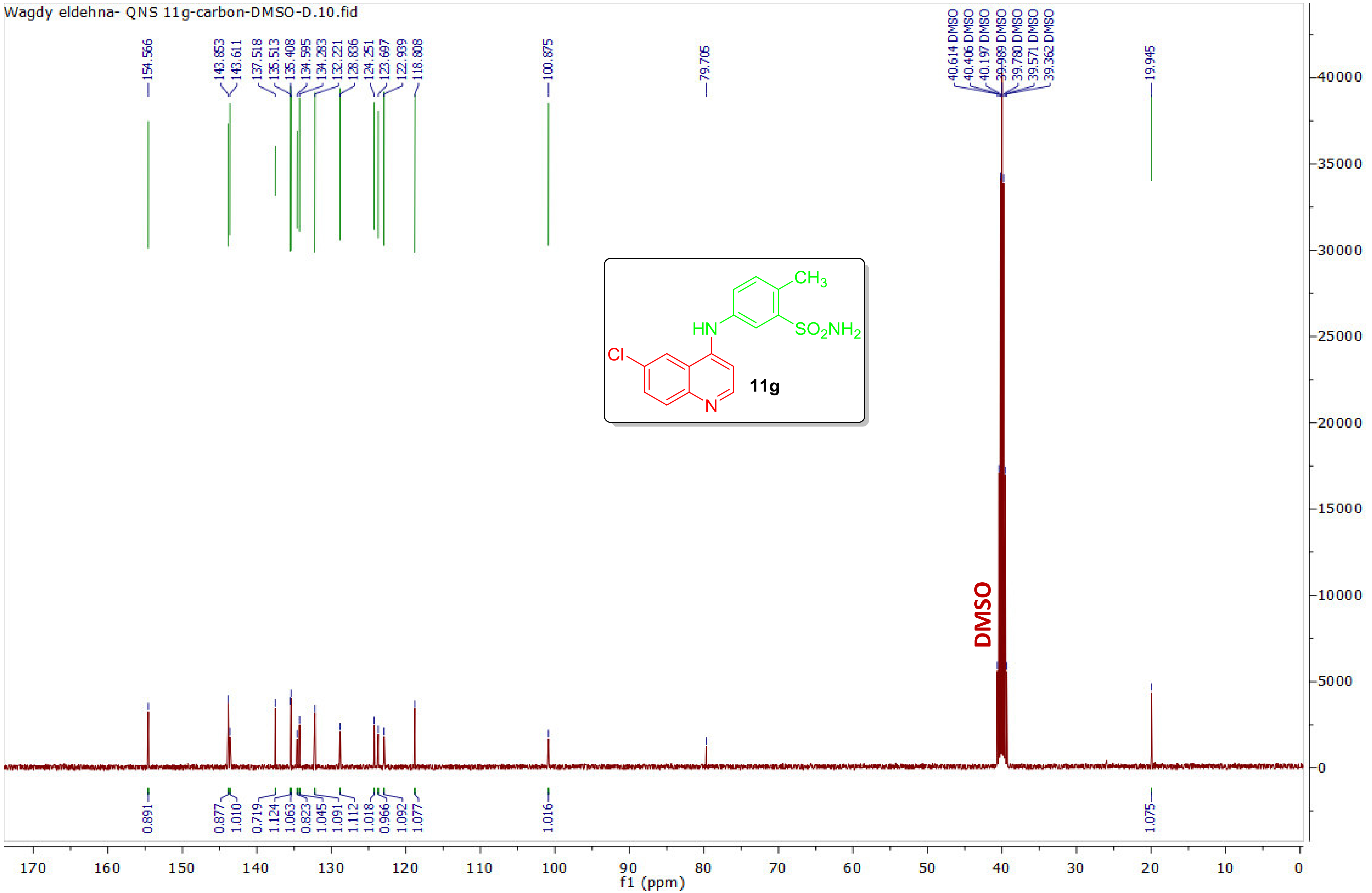
Wagdy eldehna- QNS 11g-carbon-DMSO-D.10.fid
154.566
143.853
143.611
137.518
135.513
135.408
134.595
134.283
132.221
128.836
124.251
123.697
122.939
118.808
100.875
79.705
40.614 DMSO
40.406 DMSO
40.197 DMSO
39.989 DMSO
39.780 DMSO
39.571 DMSO
39.362 DMSO
19.945
CH3
HN
SO2NH2
Cl
N
11g
DMSO
f1 (ppm)

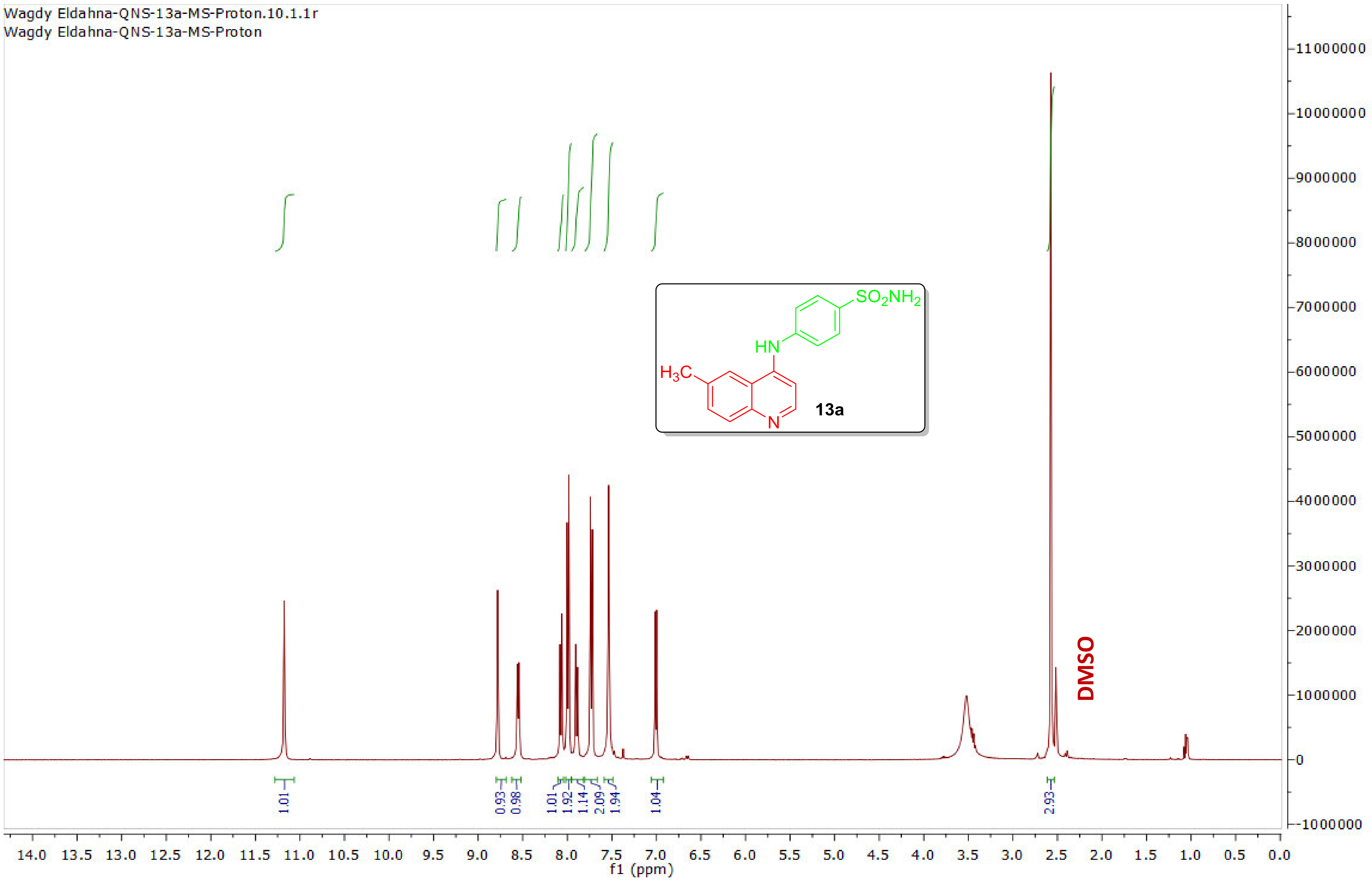
Wagdy Eldahna-QNS-13a-MS-Proton.10.1.1r
Wagdy Eldahna-QNS-13a-MS-Proton
SO2NH2
HN
H3C
N
13a
DMSO
1.01
0.93
0.98
1.01
1.92
1.14
2.09
1.94
1.04
2.93
f1 (ppm)

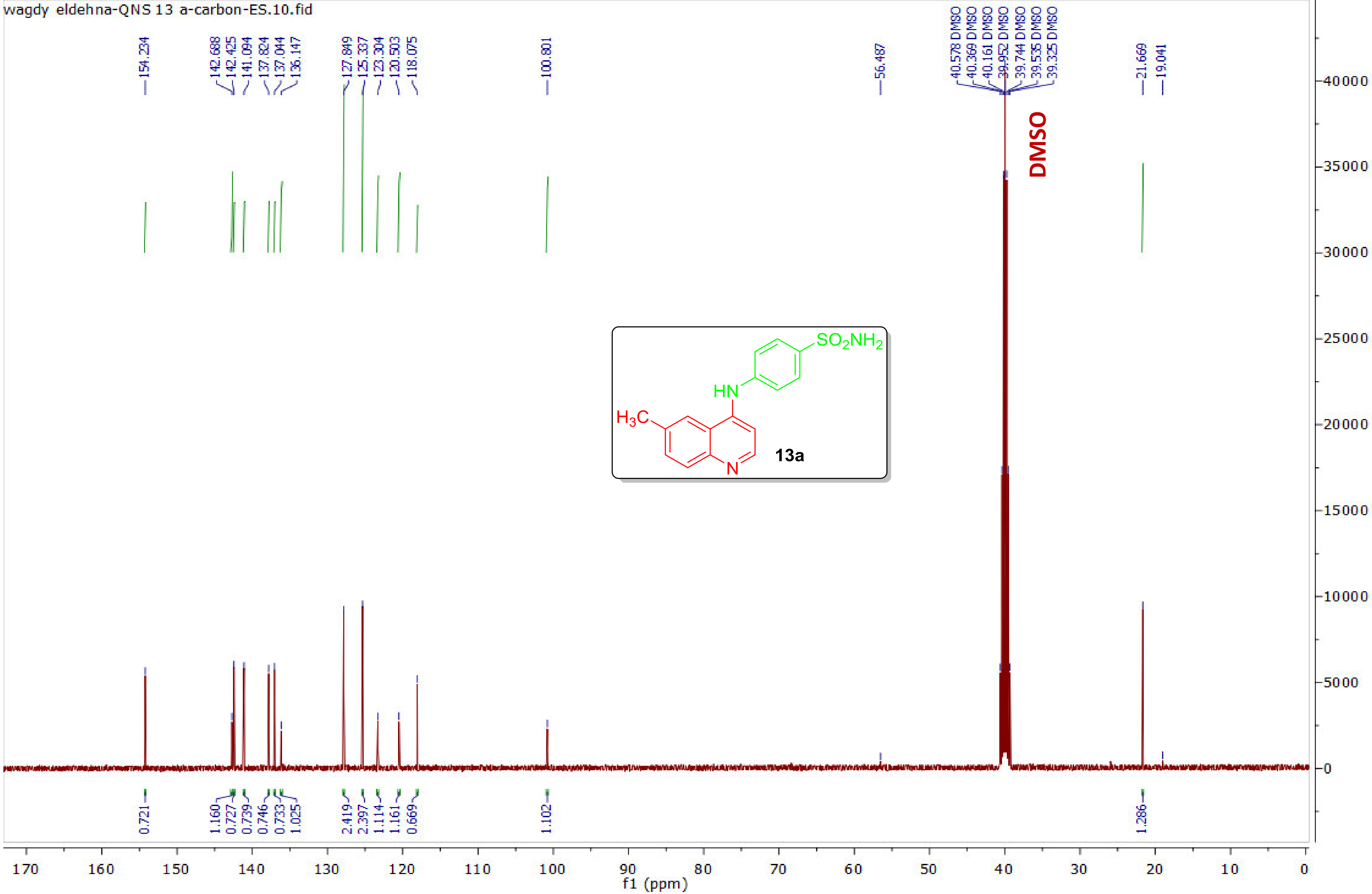
wagdy eldehna-QNS 13 a-carbon-ES.10.fid
154.234
142.688
142.425
141.094
137.824
137.044
136.147
127.849
125.337
123.304
120.503
118.075
100.801
56.487
40.578 DMSO
40.369 DMSO
40.161 DMSO
39.952 DMSO
39.744 DMSO
39.535 DMSO
39.325 DMSO
21.669
19.041
DMSO
SO2NH2
HN
H3C
N
13a
f1 (ppm)

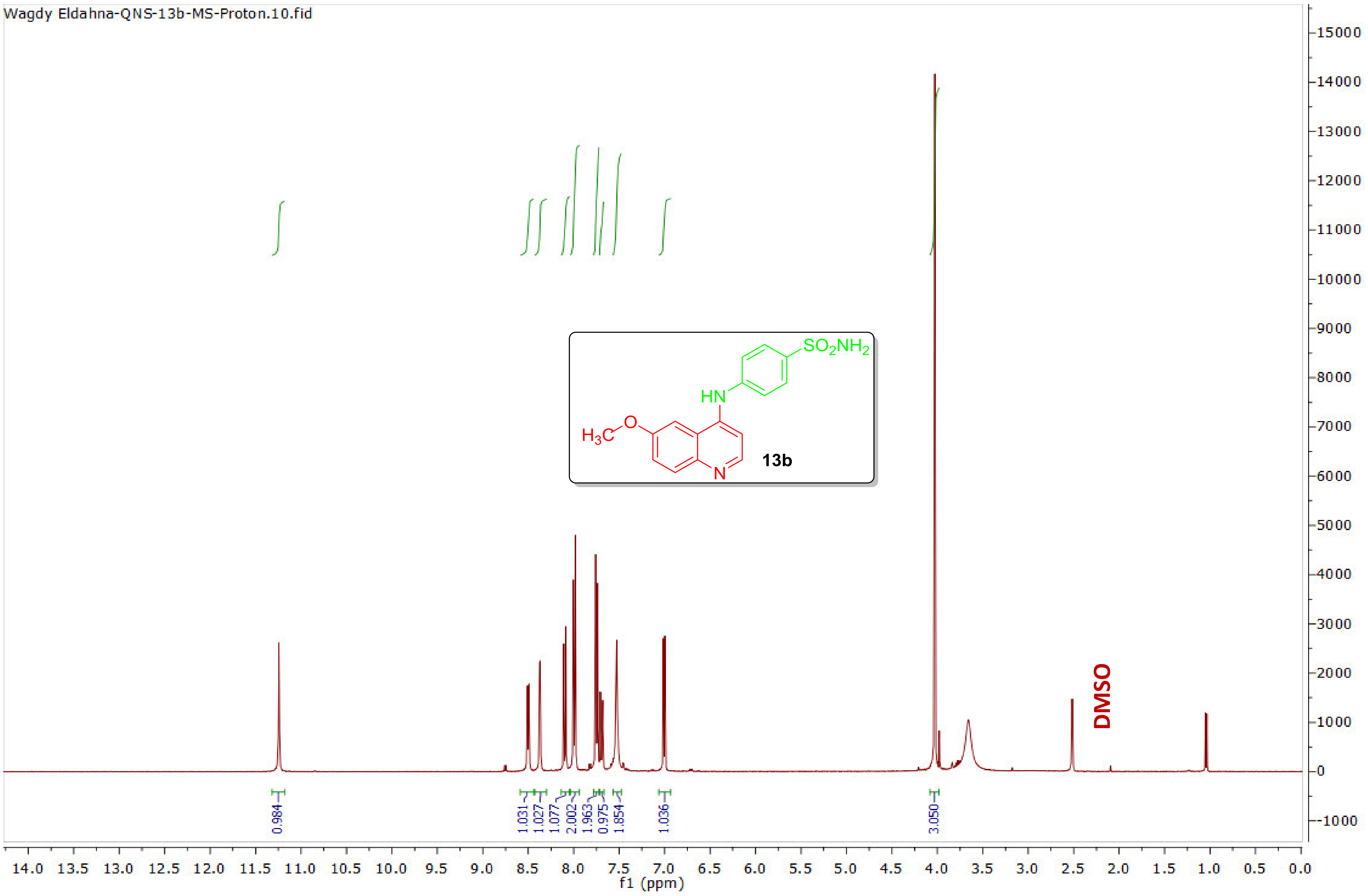

Wagdy Eldahna-QNS-13b-MS-Proton.10.fid
SO2NH2
HN
H3C
O
N
13b
DMSO
0.984
1.031
1.027
1.077
2.002
1.963
0.975
1.854
1.036
3.050
14.0 13.5 13.0 12.5 12.0 11.5 11.0 10.5 10.0 9.5 9.0 8.5 8.0 7.5 7.0 6.5 6.0 5.5 5.0 4.5 4.0 3.5 3.0 2.5 2.0 1.5 1.0 0.5 0.0
f1 (ppm)
15000
14000
13000
12000
11000
10000
9000
8000
7000
6000
5000
4000
3000
2000
1000
0
-1000

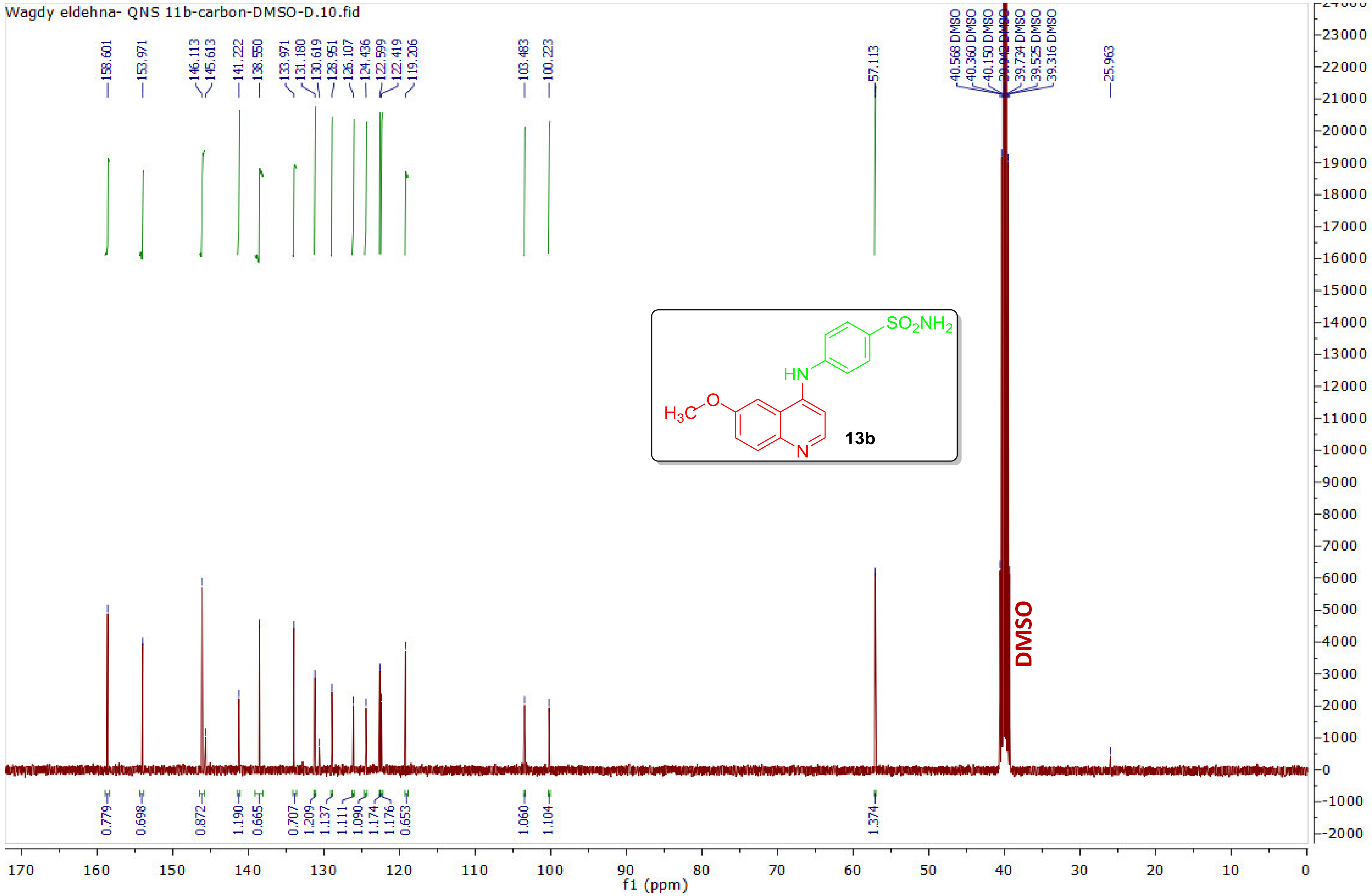
Wagdy eldehna- QNS 11b-carbon-DMSO-D.10.fid
158.601
153.971
146.113
145.613
141.222
138.550
133.971
131.180
130.619
128.951
126.107
124.436
122.599
122.419
119.206
103.483
100.223
57.113
40.568 DMSO
40.360 DMSO
40.150 DMSO
39.734 DMSO
39.525 DMSO
39.316 DMSO
25.963
SO2NH2
HN
H3C
O
N
13b
DMSO
0.779
0.698
0.872
1.190
0.665
0.707
1.209
1.137
1.111
1.090
1.174
1.176
0.653
1.060
1.104
1.374
f1 (ppm)

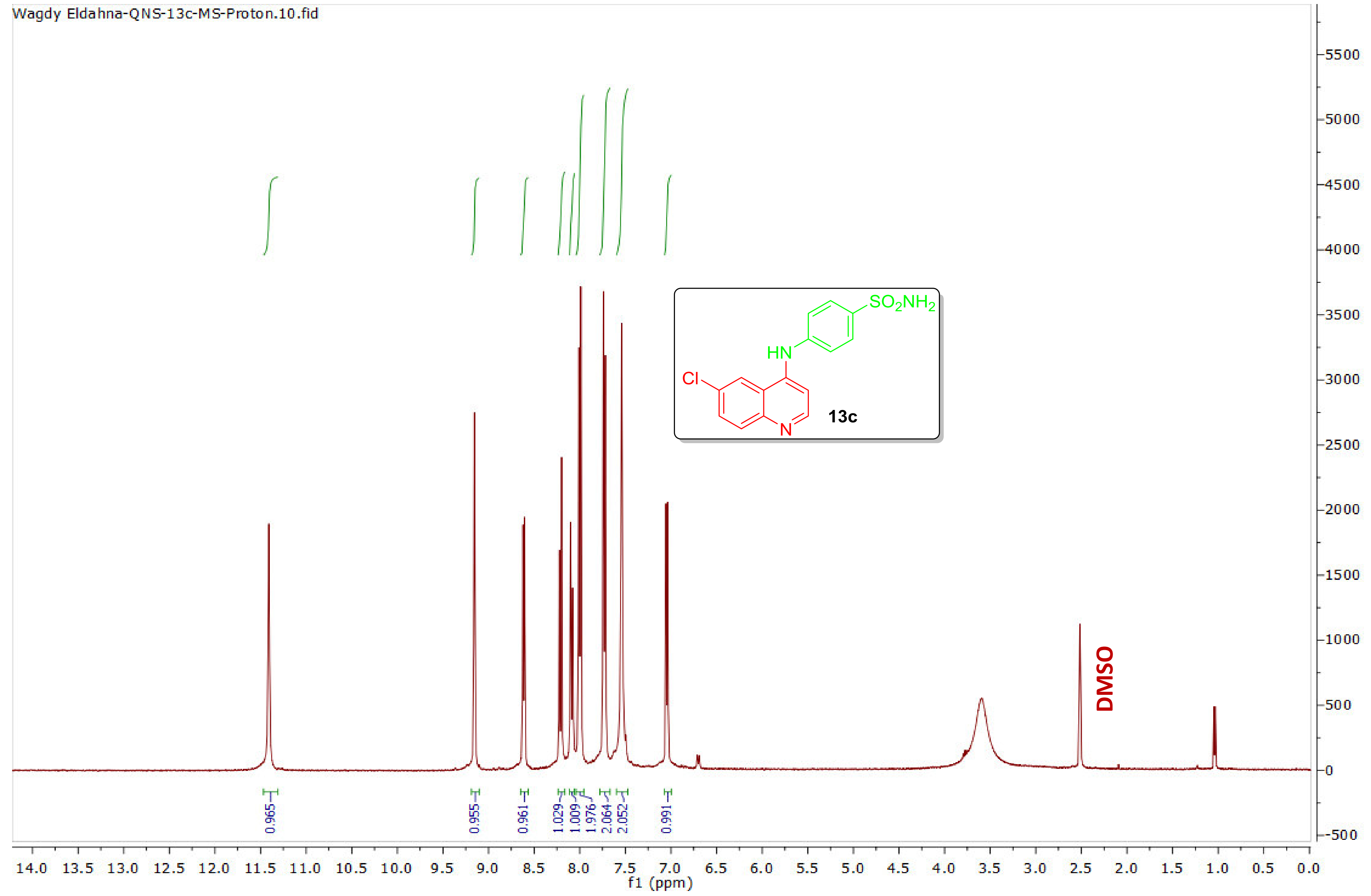

Wagdy Eldahna-QNS-13c-MS-Proton.10.fid
Cl
HN
SO2NH2
N
13c
DMSO
0.965
0.955
0.961
1.029
1.009
1.976
2.064
2.052
0.991
f1 (ppm)

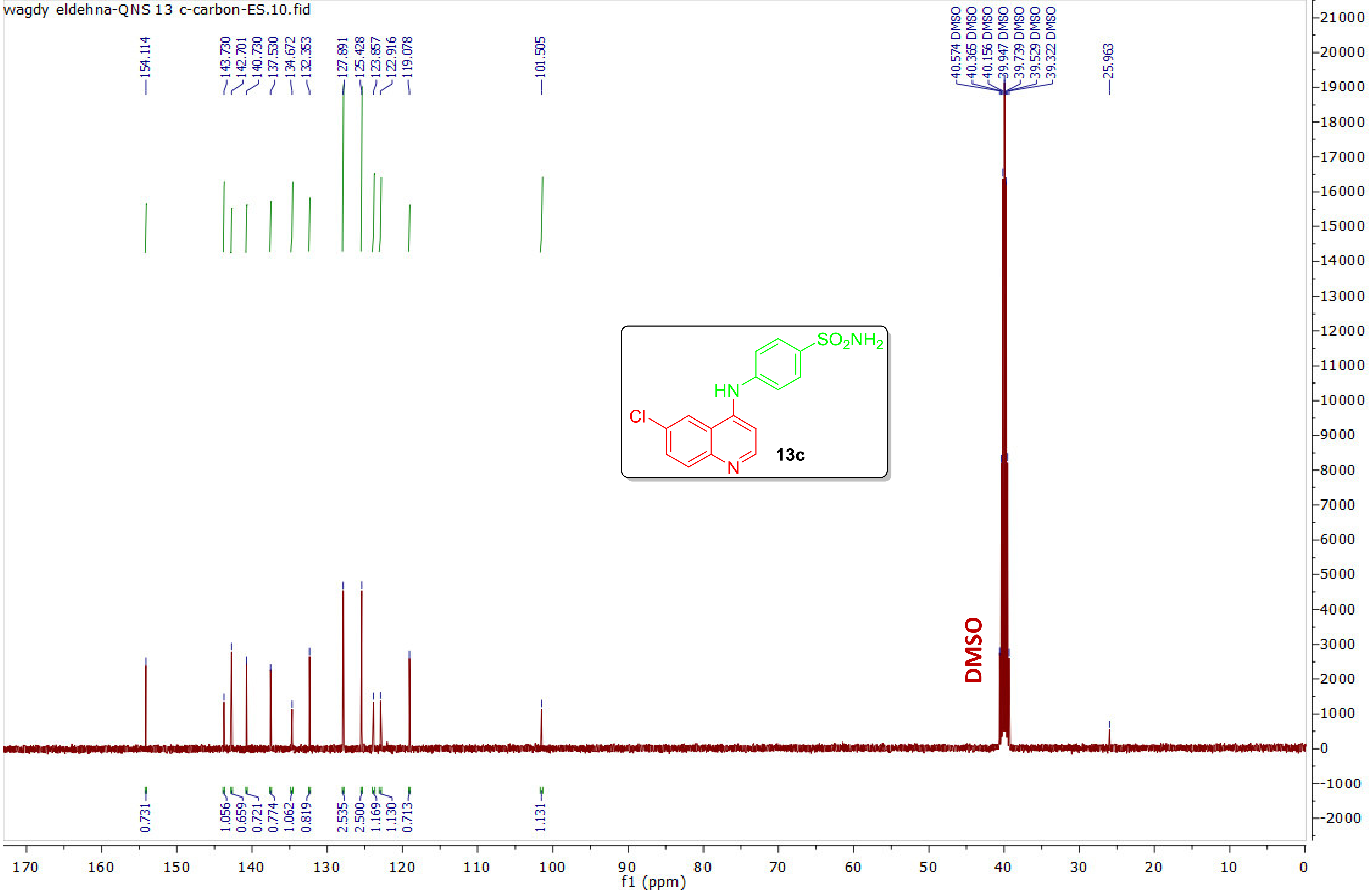
wagdy eldehna-QNS 13 c-carbon-ES.10.fid
154.114
143.730
142.701
140.730
137.530
134.672
132.353
127.891
125.428
123.857
122.916
119.078
101.505
40.574 DMSO
40.365 DMSO
40.156 DMSO
39.947 DMSO
39.739 DMSO
39.529 DMSO
39.322 DMSO
25.963
SO2NH2
HN
Cl
N
13c
DMSO
0.731
1.056
0.659
0.721
0.774
1.062
0.819
2.535
2.500
1.169
1.130
0.713
1.131
170 160 150 140 130 120 110 100 90 80 70 60 50 40 30 20 10 0
f1 (ppm)
21000 20000 19000 18000 17000 16000 15000 14000 13000 12000 11000 10000 9000 8000 7000 6000 5000 4000 3000 2000 1000 0 -1000 -2000

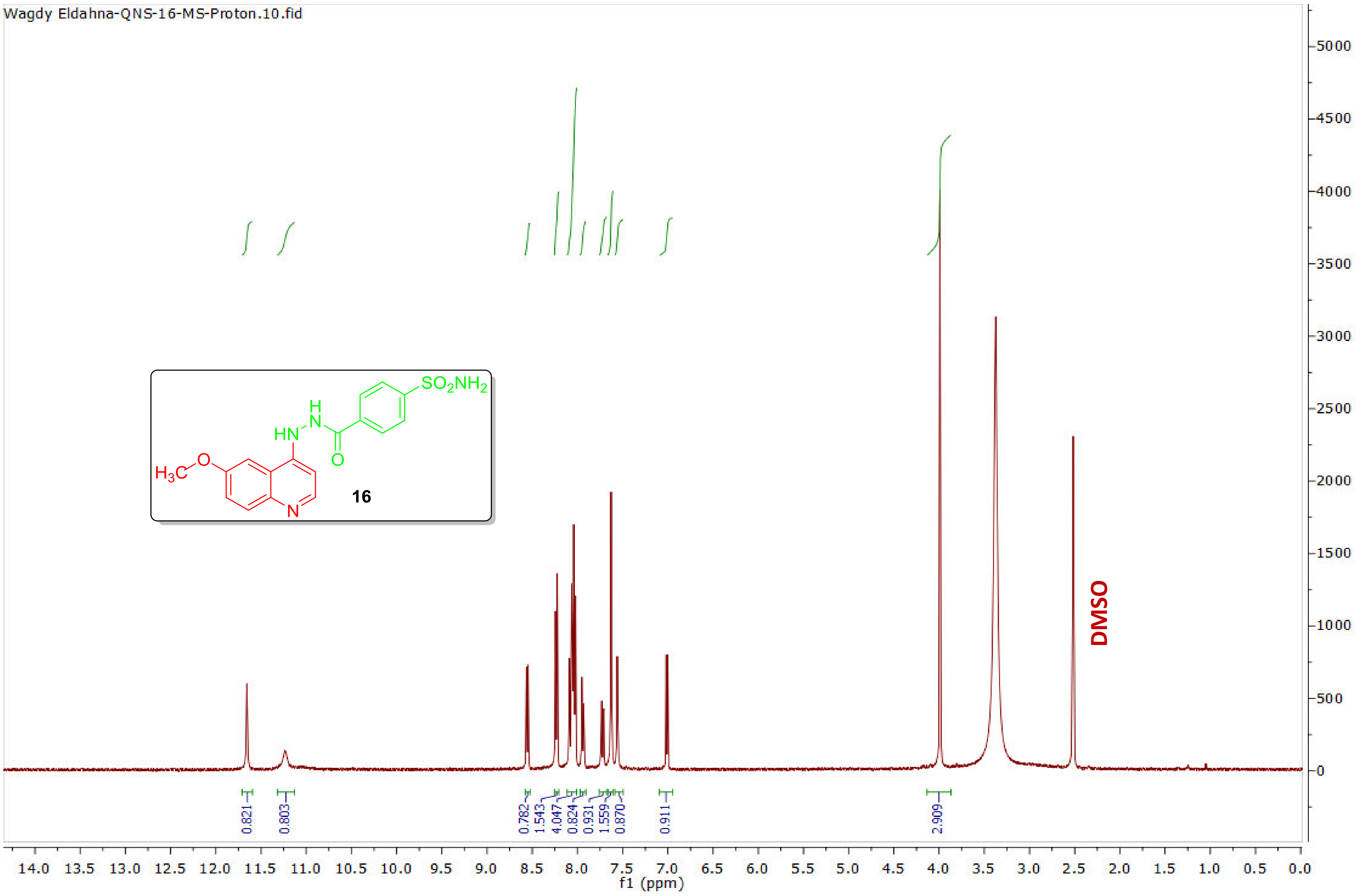
Wagdy Eldahna-QNS-16-MS-Proton.10.fid
SO2NH2
H
N
HN
O
H3C
O
N
16
DMSO
0.821
0.803
0.782
1.543
4.047
0.824
0.931
1.559
0.870
0.911
2.909
14.0 13.5 13.0 12.5 12.0 11.5 11.0 10.5 10.0 9.5 9.0 8.5 8.0 7.5 7.0 6.5 6.0 5.5 5.0 4.5 4.0 3.5 3.0 2.5 2.0 1.5 1.0 0.5 0.0
f1 (ppm)
5000
4500
4000
3500
3000
2500
2000
1500
1000
500
0

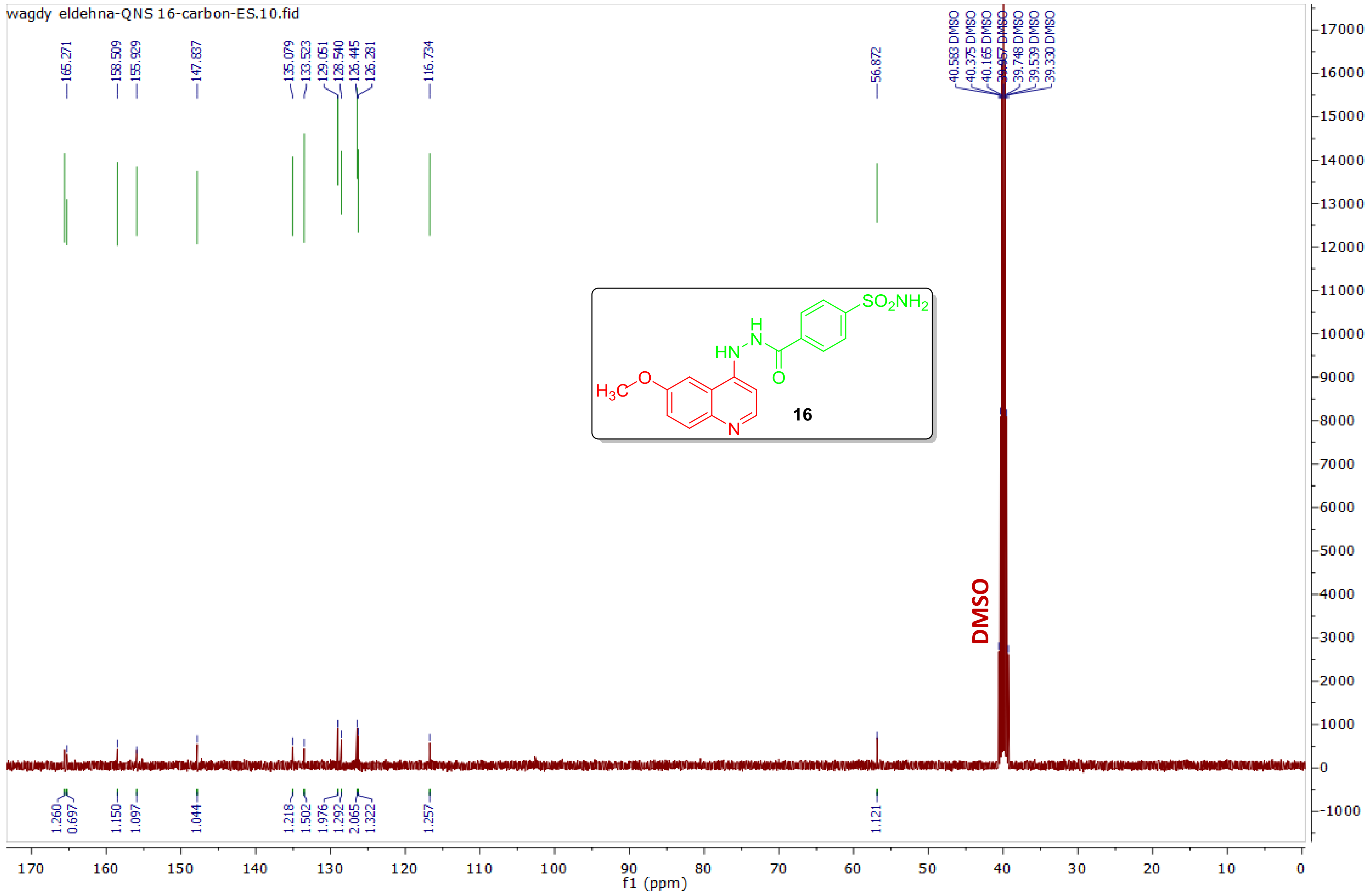
wagdy eldehna-QNS 16-carbon-ES.10.fid
165.271
158.509
155.929
147.837
135.079
133.523
129.051
128.540
126.445
126.281
116.734
56.872
40.583 DMSO
40.375 DMSO
40.165 DMSO
39.957 DMSO
39.748 DMSO
39.539 DMSO
39.330 DMSO
SO2NH2
H
N
HN
O
H3C
O
N
16
DMSO
1.260
0.697
1.150
1.097
1.044
1.218
1.502
1.976
1.292
2.065
1.322
1.257
1.121
f1 (ppm)
